# Supplementary material for: LATS1 controls CTCF chromatin occupancy and hormonal response of 3D-grown breast cancer cells
Source: EMBO J. 2024 Apr 2;43(9):5. doi: 10.1038/s44318-024-00080-x (PMC11066098; doi:10.1038/s44318-024-00080-x)
Supplement: Supplementary file 1 — Appendix [file 44318_2024_80_MOESM1_ESM.pdf]

Appendix

LATS1 controls CTCF chromatin occupancy and hormonal response of 3D-grown breast cancer cells

Julieta Ramirez Cuellar, Roberto Ferrari, Rosario T. Sanz, Marta Valverde-Santiago, Judith Garcia Garcia, A. Silvina Nacht, David Castillo, Francois Le Dily, Maria Victoria Neguembor, Marco Malatesta, Sarah Bonnin, Marc A. Marti-Renom, Miguel Beato and Guillermo P. Vicent

Table of contents

Pages 1-21: Appendix Figures S1-S15

Pages 22-25: Extended Bioinformatics methods

Pages 26-27: Appendix References

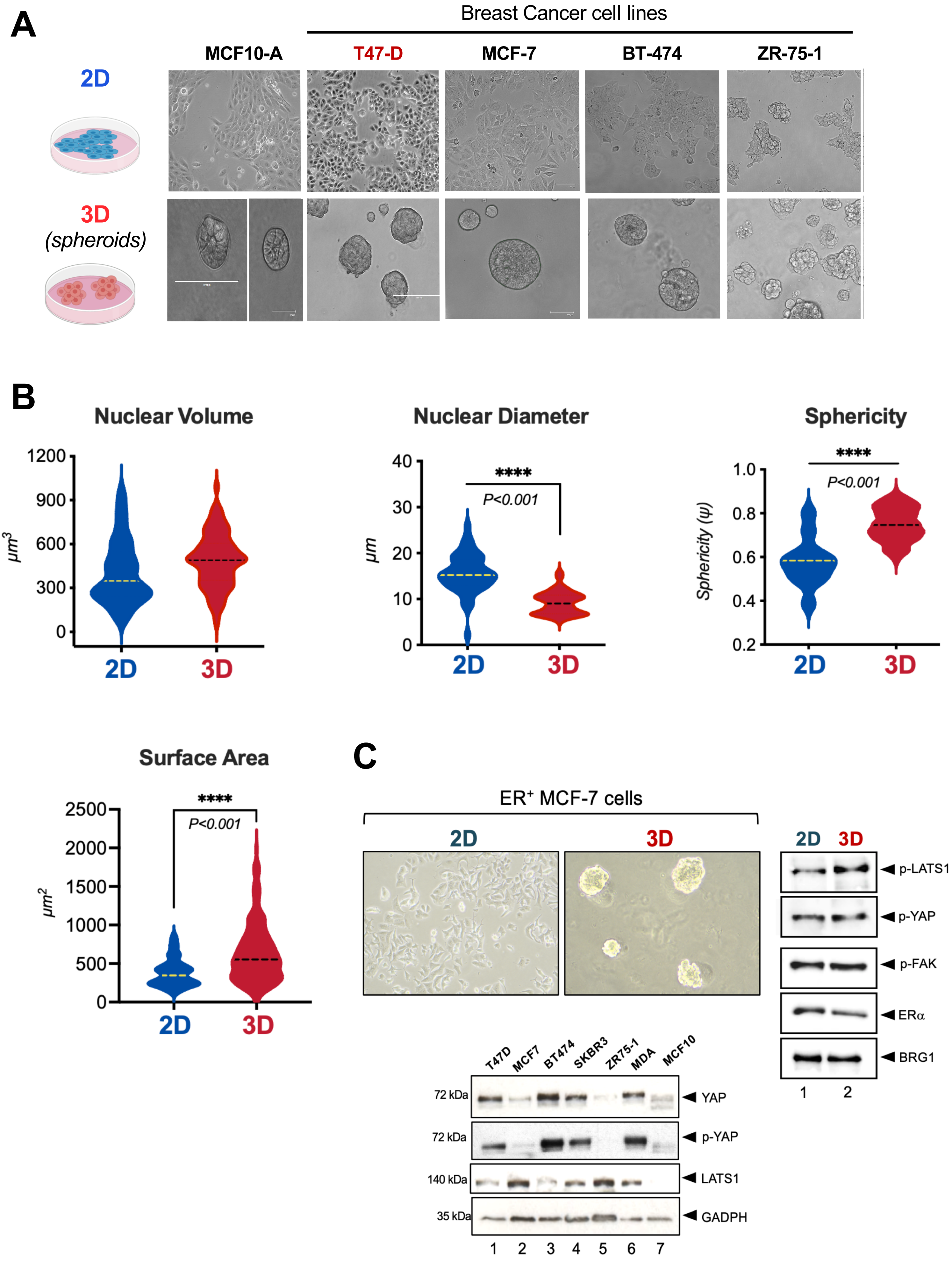

Appendix Figure S1
Ramirez et al.

Appendix Figure S1***. Breast cell lines grown in 3D culture embedded in Matrigel***
Comparison of different breast cell lines grown on the plastic dish (2D) or as 3D spheroids in Matrigel. All cell lines in 3D can create defined spheres except for ZR75 which forms grape-like structures. Scale bar: 50 µm. To improve the comparison between cell lines, the microscopy images of MCF-10A and T47D cells, grown as monolayer or as spheroids, are the same to those previously displayed in Figure 1A. *Physical properties of 3D cells.* The nuclei of the 3D cells measured with Imaris/ImageJ showed a larger volume accompanied by an increase of sphericity and larger surface area compared to 2D nuclei. **C.** MCF-7 cells were grown in 2D and 3D and the levels of p-LATS, p-YAP, p-FAK, ER and BRG1 was determined (right panel). High heterogeneity in YAP, p-YAP and LATS was found between different breast tumoral (lanes 1-6) and non tumoral (lane 7) cell lines.

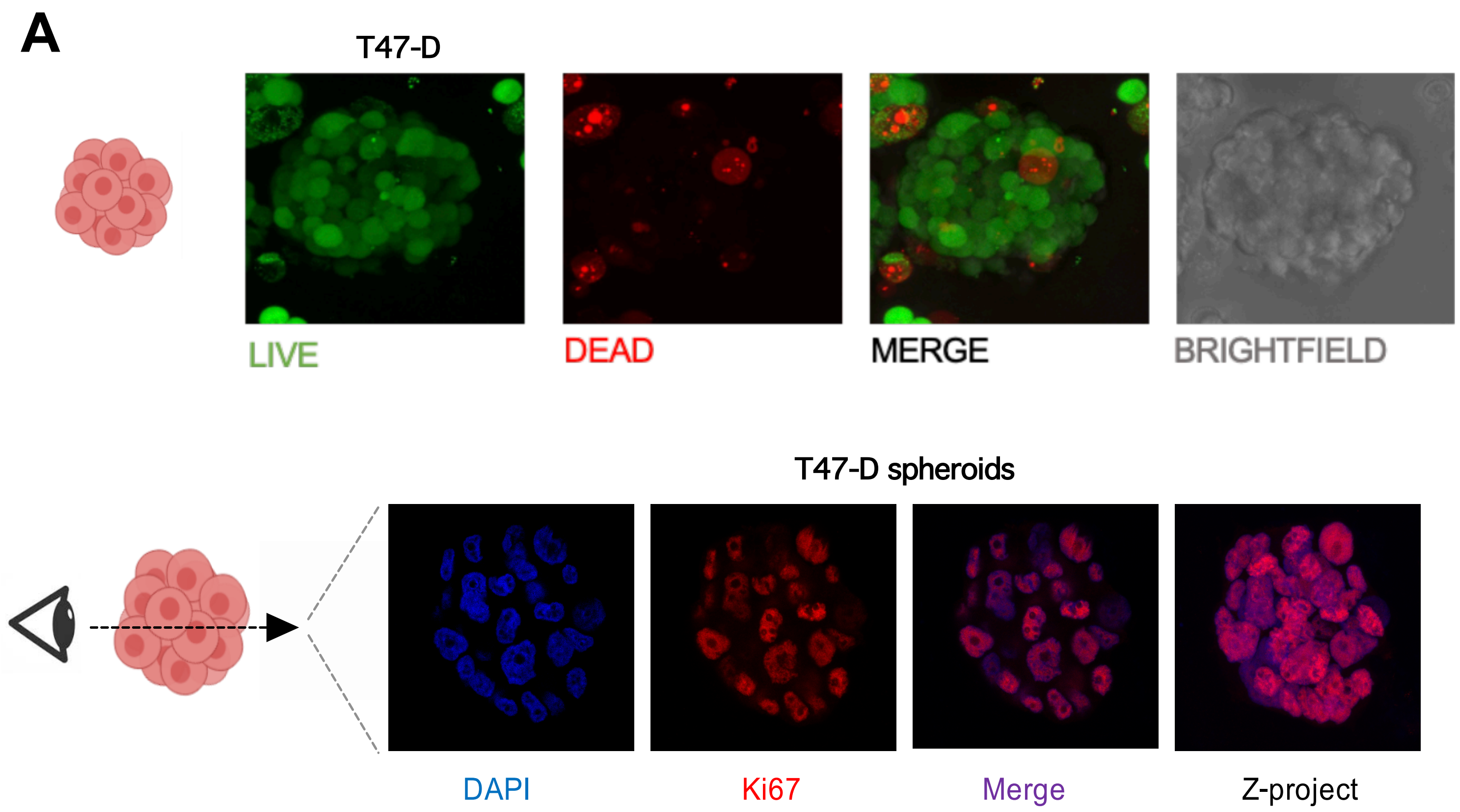

Appendix Figure S2. ***Cell proliferation in T47D spheroids***
A. 3D T47D spheroid stained for live/dead cells after 10 days in culture. Apoptotic cores are rather small, sporadic and not predominant in the core of the spheroid. Bottom: Slice of a 10-day spheroid that was stained for the proliferative marker Ki-67 and overlapped with DAPI. Images were acquired on a Leica SP8-STED confocal laser-scanning microscope using the software Leica**.** Application Suite X with filters 488 (green) and 546 (red).

Appendix Figure S2
Ramirez et al.

A

GO: Biological process	Activated	Repressed
Term Name	p_adj	p_adj
biological adhesion	1.329×10^{-2}	9.544×10^{-16}
cell adhesion	1.105×10^{-2}	1.433×10^{-14}
multicellular organism development	2.632×10^{-8}	2.732×10^{-13}
generation of neurons	8.158×10^{-11}	1.000
neurogenesis	9.481×10^{-11}	1.000
nervous system development	7.917×10^{-9}	1.000
actin filament-based process	1.000	4.081×10^{-7}
response to wounding	1.000	5.117×10^{-7}
regulation of neurogenesis	1.882×10^{-6}	1.000
regulation of cell development	1.885×10^{-6}	1.000
axonogenesis	3.274×10^{-6}	1.000
positive regulation of cell migration	1.000	3.687×10^{-6}
positive regulation of cell adhesion	1.000	4.530×10^{-6}
regulation of cell differentiation	5.195×10^{-6}	2.721×10^{-4}
neuron projection development	6.385×10^{-6}	1.000
axon development	6.450×10^{-6}	1.000

GO: Cellular Component	Activated	Repressed
Term Name	p_adj	p_adj
anchoring junction	1.000	2.102×10^{-13}
focal adhesion	1.000	2.120×10^{-10}
extracellular region	8.600×10^{-2}	2.810×10^{-10}
cell-substrate junction	1.000	3.898×10^{-10}
cell junction	4.029×10^{-2}	4.072×10^{-9}
cell periphery	1.477×10^{-3}	1.112×10^{-8}
plasma membrane	1.217×10^{-3}	4.337×10^{-8}
contractile fiber	1.000	5.826×10^{-6}
actin cytoskeleton	1.000	8.362×10^{-6}

B

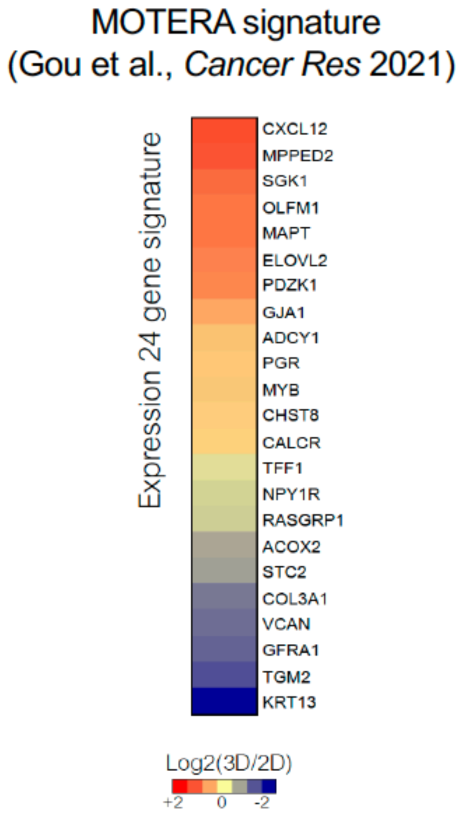

Appendix Figure S3 ***Gene Ontology of Biological Process (BP) and Cellular Component (CC) differentially regulated in 3D cells.*** **A.** Using DEA results, GO was performed for Biological Process (upper panel) and Cellular component (lower panel). The main terms enriched are associated with cell adhesion processes, cell structure and regulation of neurogenesis. **B.** Mutant or Translocated Estrogen Receptor Alpha (MOTERA) signature heatmap for our RNA-seq experiments of 3D versus 2D gene expression was generated recovering the log2 ratio (3Dvs2D) for each of the 24 genes belonging to the signature. Cluster 3.0 was used to generate the CDT file loaded on Java Tree View for heatmap visualization. Genes were ranked from the highest expressed to the lowest expressed.

Appendix Figure S3
Ramirez et al.

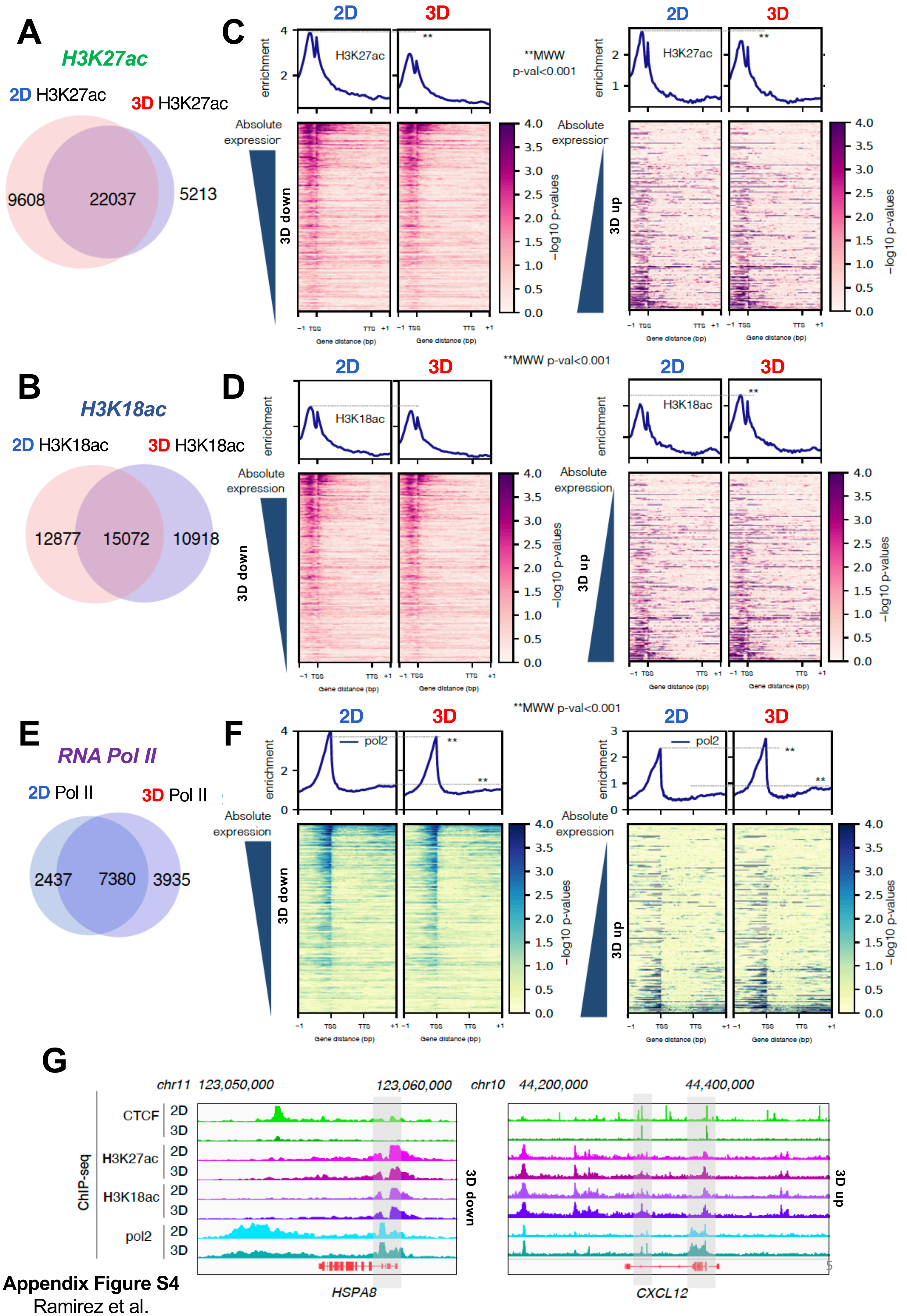

Appendix Figure S4
Ramirez et al.

Appendix Figure S4. ***Epigenetic marks and RNApol II distribution in 2D and 3D grown cells***

Venn diagrams of H3K27ac (**A**), H3K18ac (**B**) and RNApol II (**E**) assayed in 2D and 3D grown cells. The H3K27ac (**C**), H3K18ac (**D**) and RNApol II (**F**) profiles in genes repressed or activated in 3D cells (left and right panels, respectively) is shown. **G.** Genome browser view of ChIP-seq data of *HSPA8* and *CXCL12,* two representative genes of each condition.

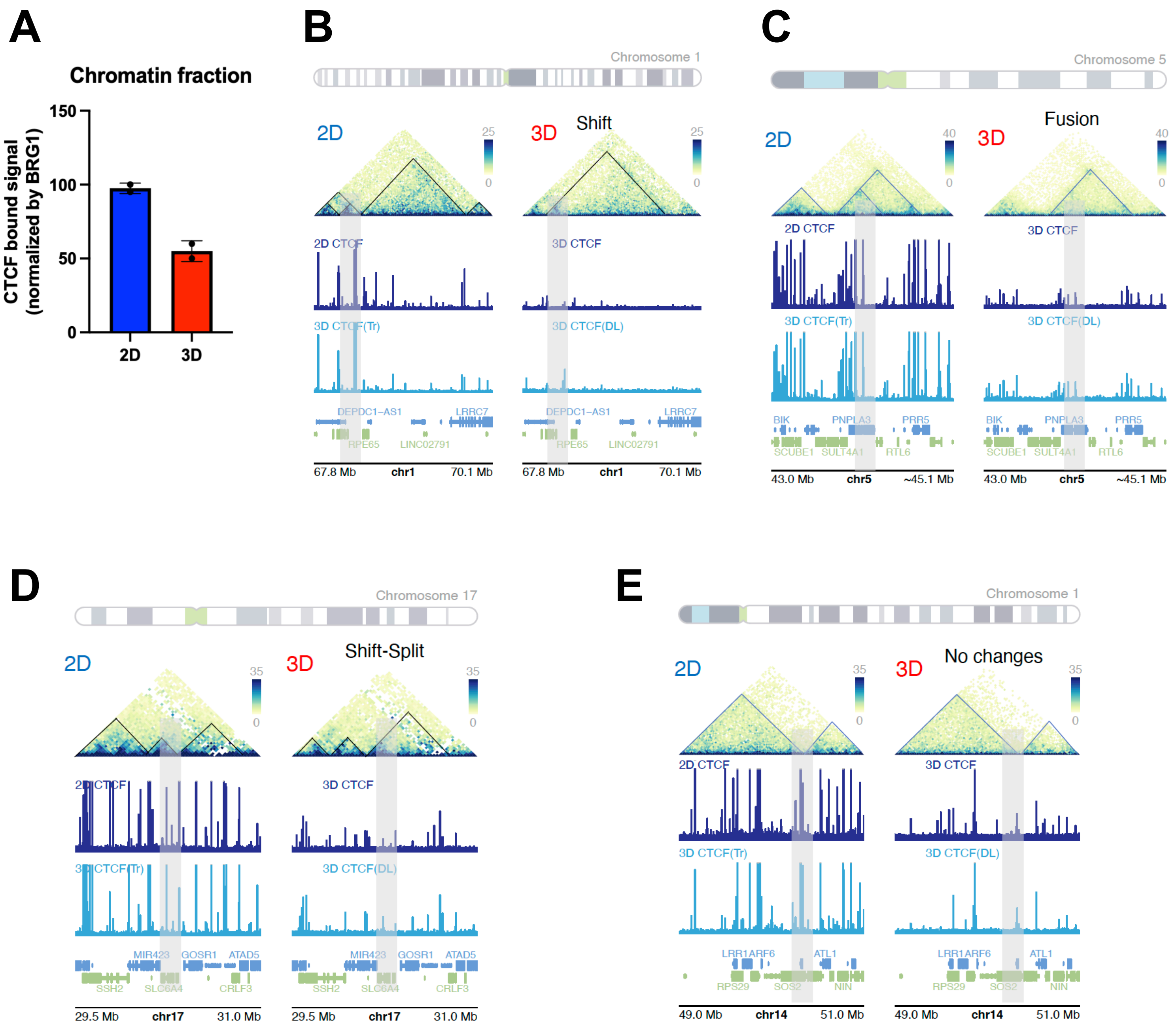

F

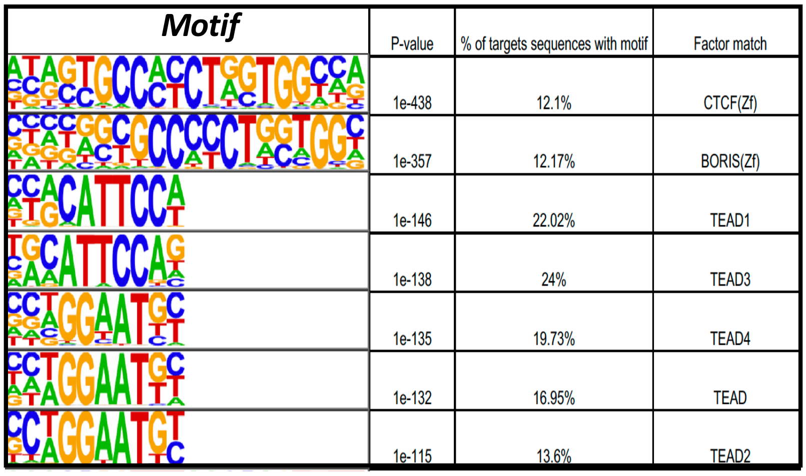

Motif	P-value	% of targets sequences with motif	Factor match
	1e-438	12.1%	CTCF(Zf)
	1e-357	12.17%	BORIS(Zf)
	1e-146	22.02%	TEAD1
	1e-138	24%	TEAD3
	1e-135	19.73%	TEAD4
	1e-132	16.95%	TEAD
	1e-115	13.6%	TEAD2

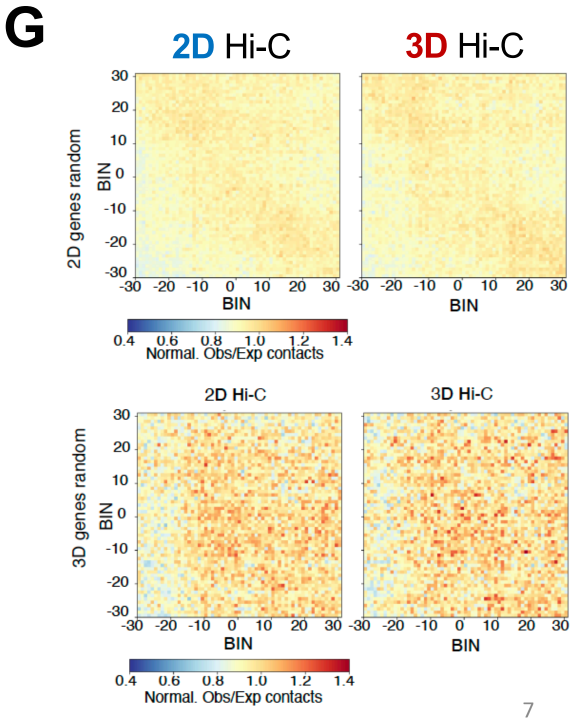

Appendix Figure S5
Ramirez et al.

Appendix Figure S5. ***Loss of CTCF binding detected in 3D cells is not exclusively found at TAD fusions***

A. T47D cells grown in 2D and 3D conditions were subjected to cell fractionation experiments (Mendez and Stillman, 2000). The chromatin fractions obtained in both conditions were run in polyacrylamide gels and western blot for CTCF was performed. The CTCF bound to chromatin was quantified using ImageJ software and the signal was corrected by the presence of BRG1. **B.** Loss of CTCF binding overlaps with: i) 3D-exclusive TAD split and DEG (green), ii) TAD fusions (blue), iii) unchanged TAD borders (gray) and iv) TAD fusions without any change in CTCF (purple). **C.** Loss of CTCF overlaps with TAD shifts and DEG (yellow) as well as with unchanged TAD borders (gray). **D.** Loss of CTCF binding without any changes in TAD border is shown (gray). **E.** Motif analysis of the ATAC-seq peaks: HOMER motif analysis identifies significant enrichment of the CTCF, CTCFL and TEAD motifs in ATAC-seq peaks with decreased signal in 3D. **F.** Hi-C explorer aggregate plots. Long-distance interactions among 3D and 2D random genes. The genomic coordinates of the random genes are centered between half the number of bins and the other half number of bins. Plotted are the submatrices of the aggregated contact frequency for 20 bins (1.5 kb bin size, 35 kb in total) in both upstream and downstream directions. Color bar scale with increasing red shades of color stands for higher contact frequency.

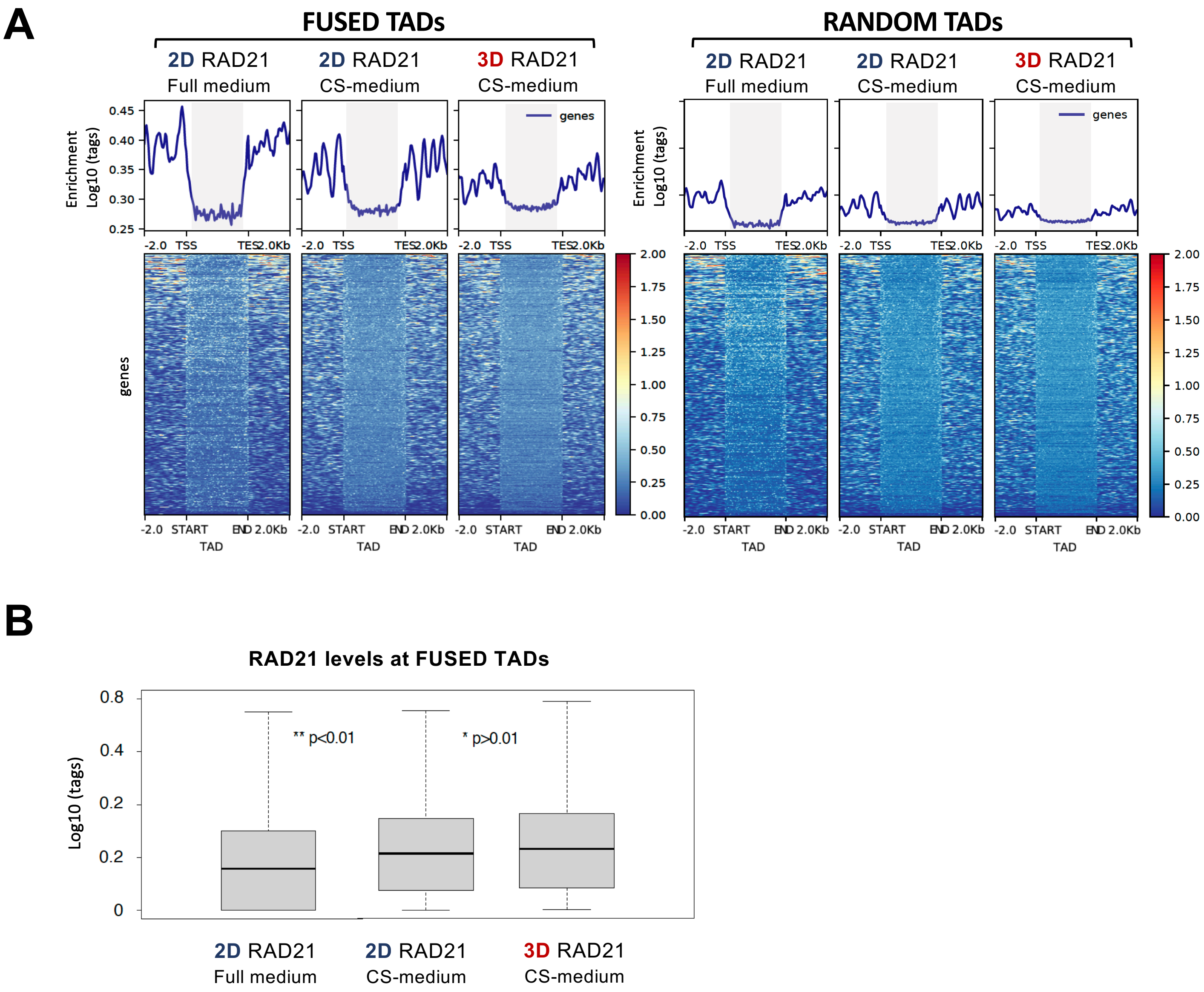

Appendix Figure S6. ***RAD21 distribution in 2D and 3D grown T47D cells***
A. Heat maps of the cohesin component RAD21 ChIP-seq data around 3D-fused (left panel) and random Topological Associated Domains (TADs) (right panel). **B.** Box plots of the Log10 (tags) around each category. 2D: monolayer, 3D: spheroids, Full medium, CS: medium containing 10% charcolized serum without phenol red.

Appendix Figure S6
Ramirez et al.

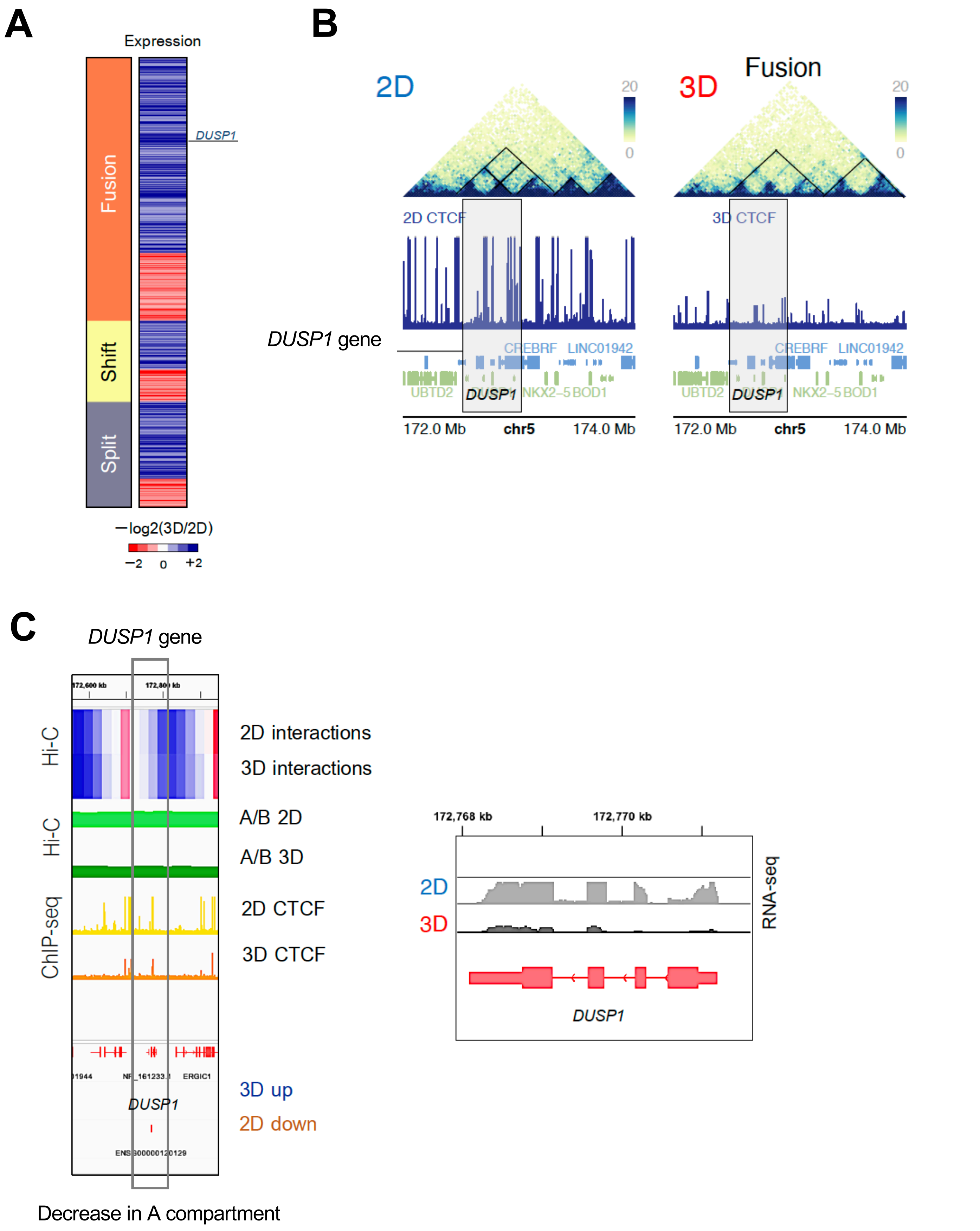

Appendix Figure S7. ***CTCF displacement particularly impacts in 3D genes***
CTCF displacement impacts in the 3D down gene *DUSP1*, by changing their contact environment through TADs fusion as shown (A-C). *DUSP1* expression is associated with an increased risk of metastasis and shorter overall survival in breast cancer.

Appendix Figure S7
Ramirez et al.

A

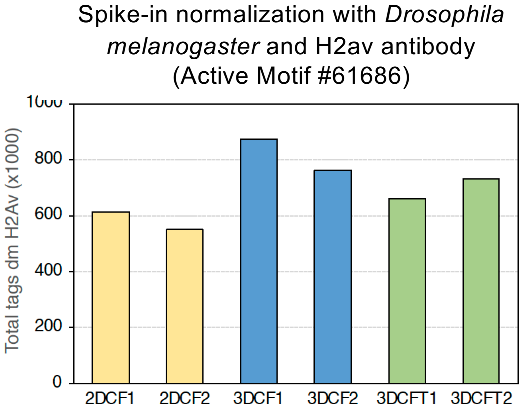

B

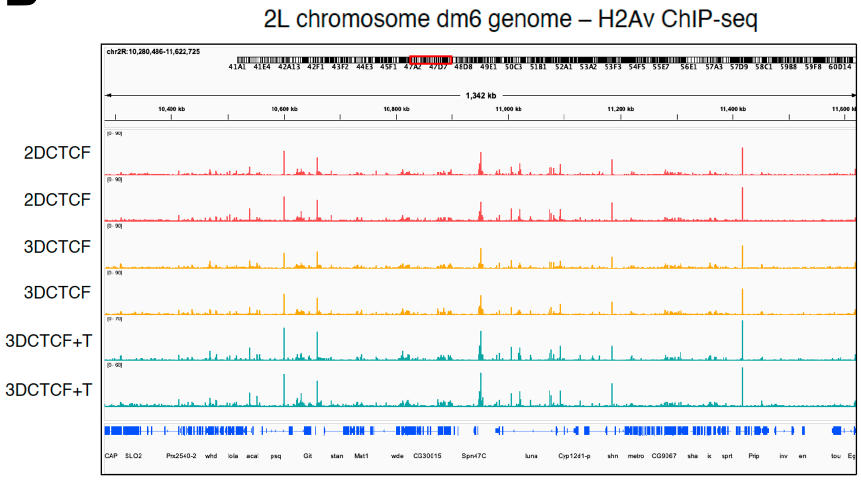

C

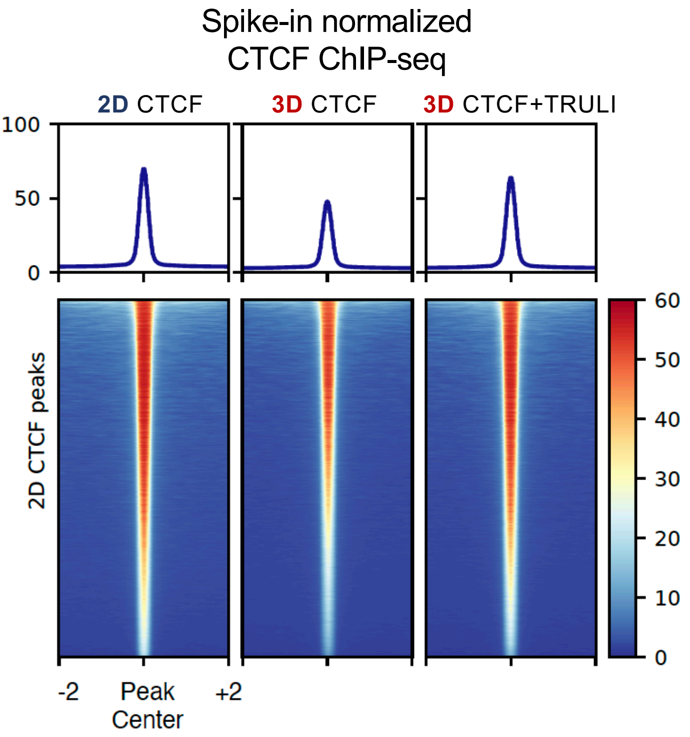

Calculated factor for tags normalization
3D/2D

**3D** CTCF rep 1	1.424887
**3D** CTCF rep 2	1.380979
**3D** CTCF+TRULI rep 1	1.078732
**3D** CTCF+TRULI rep 2	1.324471

D

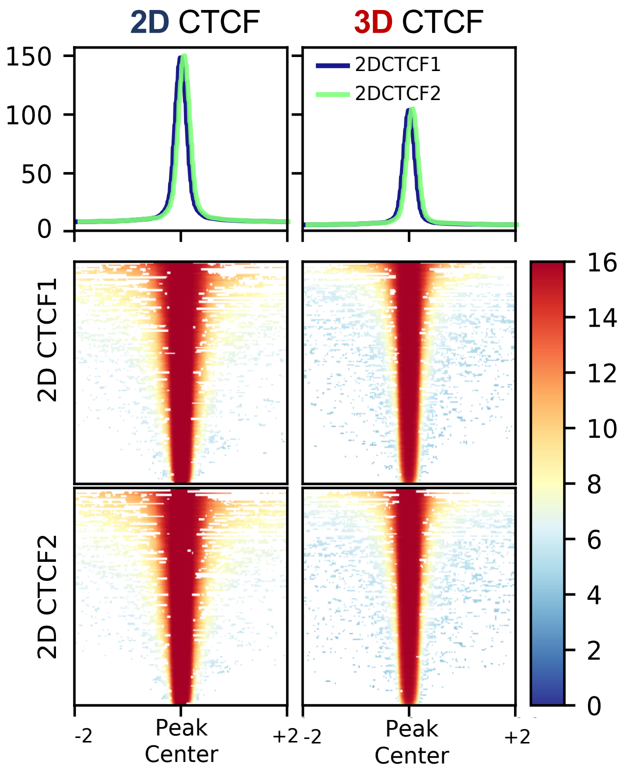

Appendix Figure S8
Ramirez et al.

Appendix Figure S8. ***CTCF binding in 2D and 3D grown T47D cells using spike-in controls***

A. Spike in normalization using Drosophila melanogaster chromatin and anti H2Av antibody (Active Motif) for CTCF ChIP-seq performed under mild conditions in 2D (yellow bars), 3D (blue bars) and 3D+TRULI (green bars) T47D cells. **B.** Snapshot of the genome browser showing the profile of H2Av ChIP-seq around the Drosophila 2L chromosome. **C.** Heatmaps of the CTCF ChIP-seq signal performed in 2D, 3D and 3D+TRULI cells and normalized using Drosophila melanogaster spike-in controls. **D.** *Heatmap of merged CFCT binding profiles*. CTCF ChIP-seq profiles from 2D and 3D grown cells have been merged to achieve an average value across replicates. BigWigMerge (UCSC genome tools) has been employed to merge all the bigwig profiles of all three replicates and generate one unique bigwig file. deepTool was implemented to compare newly generated merged bigwig to set of two replicates of 2D CTCF peaks. Significance of decrease has been measured by two-way ANOVA test.

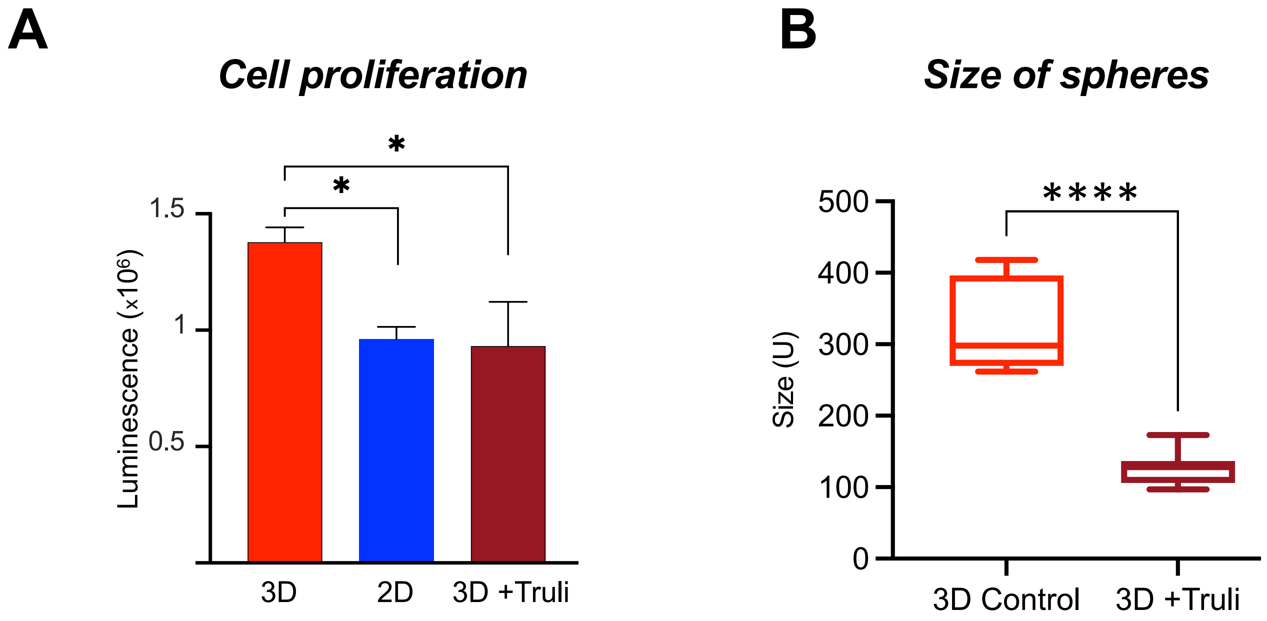

Appendix Figure S9 ***Effect of LATS1 inhibition on cell proliferation of 3D T47D cells***
Cells grown in 2D and 3D conditions and treated or not with TRULI as indicated, were assayed for cell proliferation (**A**) and sphere size measurement after 10 days of culture (**B**).

Appendix Figure S9
Ramirez et al.

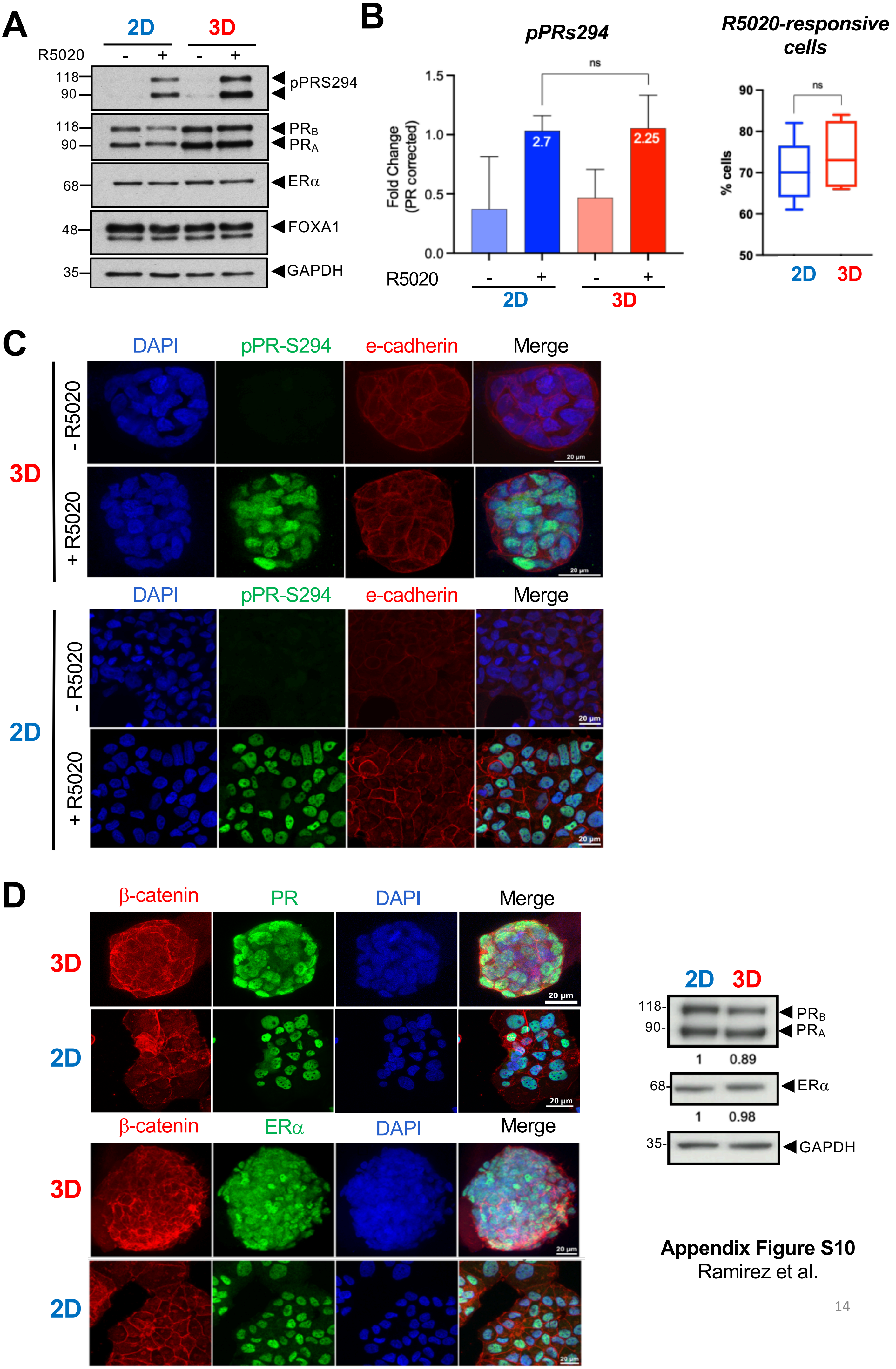

Appendix Figure S10
Ramirez et al.

Appendix Figure S10. ***Activation of Progesterone Receptor in 2D and 3D T47D cells***

Activation of Progesterone Receptor in 2D and 3D T47D cells. **A.** phosphoPR S294, total PR, ERa and FOXA1 levels were monitored in 2D and 3D cells treated or not with 10 nM R5020 for 30 min. **B.** PR activation between 2D and 3D conditions were assayed (left). The percentage of T47D cells that turned out to be responsive to hormone in both culture systems is shown. **C.** Immunostaining of 3D and 2D cells in the presence and in the absence of 10 nM of R5020 for 30 min, DAPI (blue), phospho-PR S294 (green), e-cadherin (red), and merge of all channels. **D.** PR and ERα expression in T47D cells grown under 2D and 3D conditions. Distribution of ERα, PR and β-catenin were assayed by immunofluorescence (IF) in 2D and 3D conditions. Scale bar: 20μm (left panels). Levels of PR and ERα between conditions were assessed by western blot using specific antibodies (right panel).

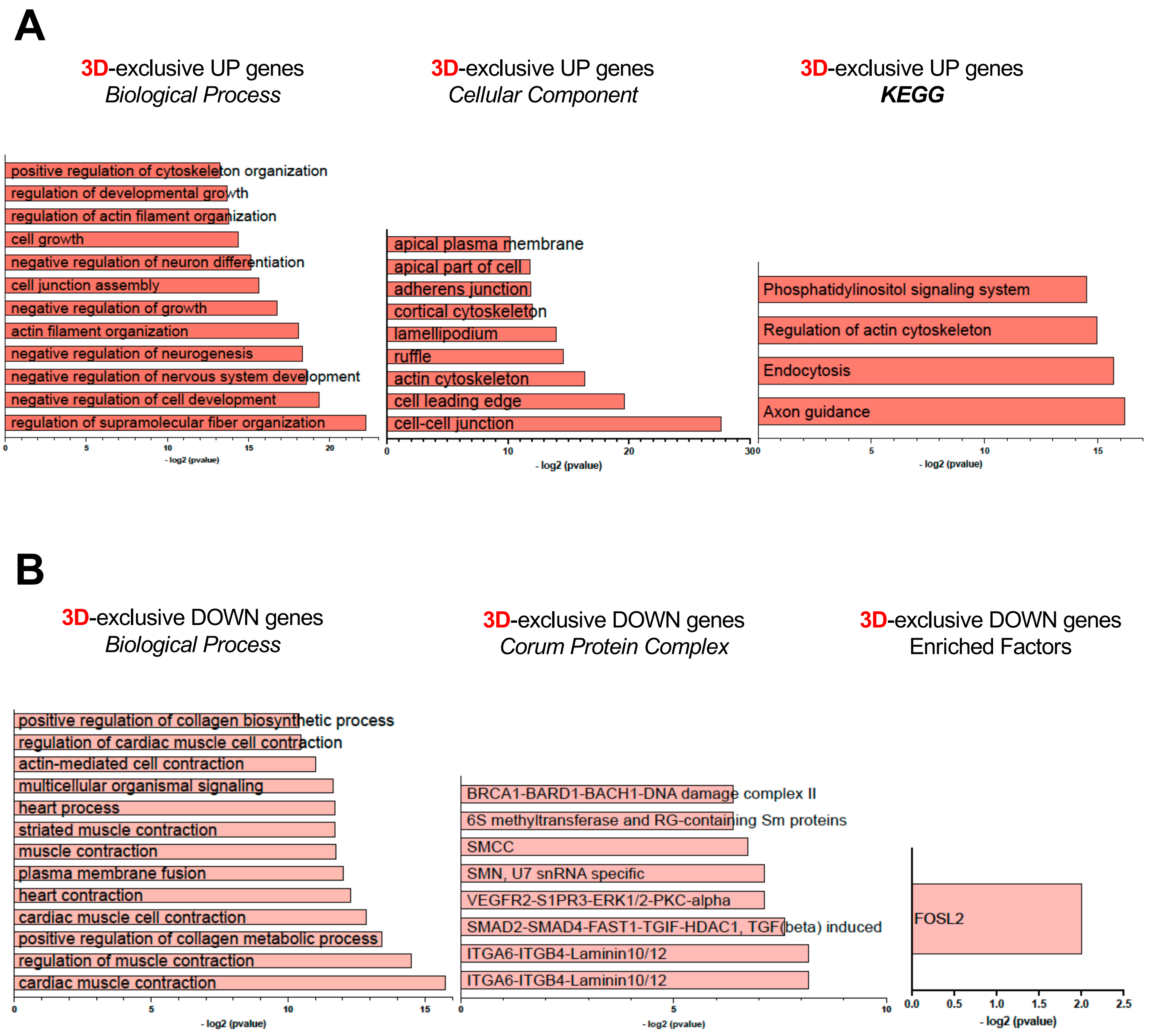

Appendix Figure S11. ***Enriched GO terms in 3D T47D cells treated with R5020***
Using DEA results (Figure 6A), GO analysis was performed on 3D differentially expressed genes. The top terms are shown. The main enriched terms for up-regulated genes are associated with *cell structure, neurogenesis* and *neuron development*, while for down-regulated genes categories are related to *cell membrane components* and *cell contraction*.

Appendix Figure S11
Ramirez et al.

A

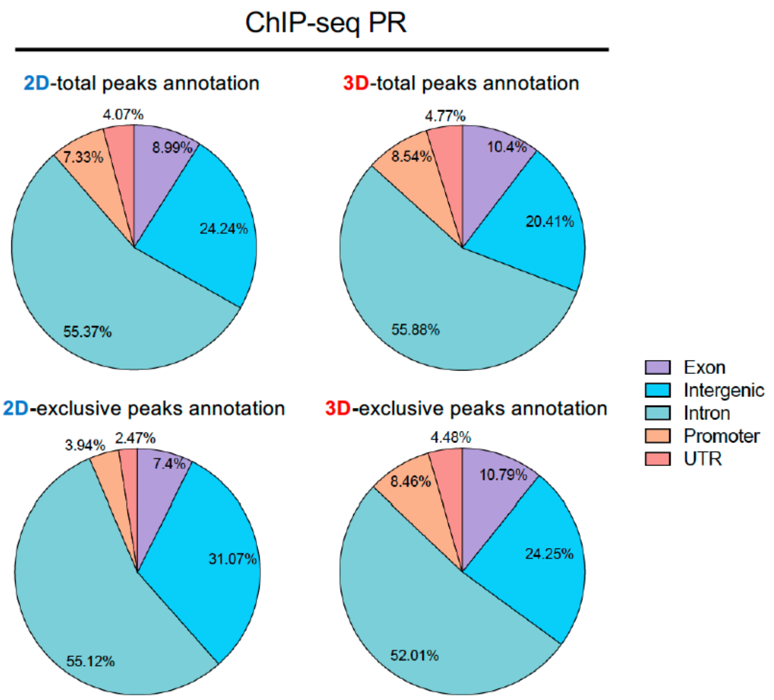

B

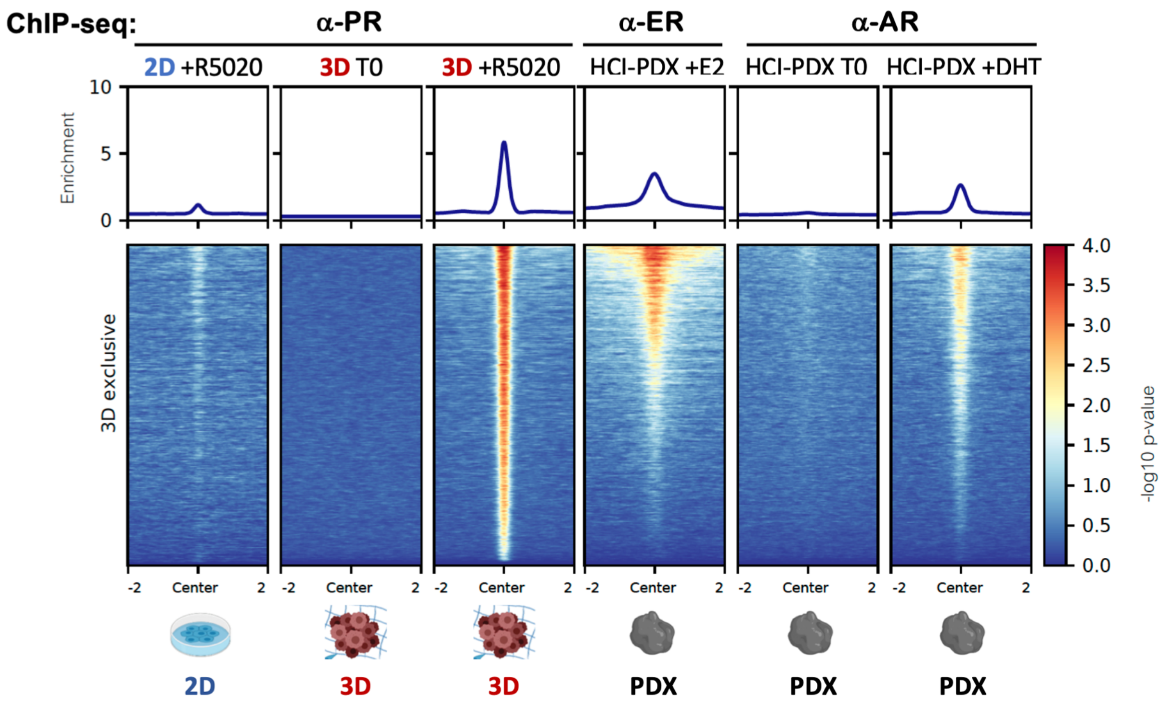

C

3D-exclusive PRBs motif enrichment

Motif	P-value	% of targets	Factor match
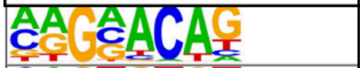	1e-939	75.45%	PR (NR)
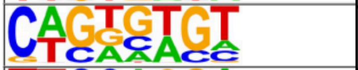	1e-696	57.59%	ZNF768
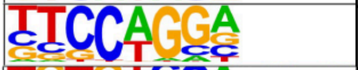	1e-520	61.60%	Bcl6
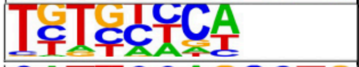	1e-370	37.80%	MAC1
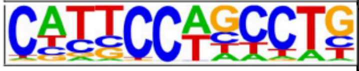	1e-81	5.53%	TEAD2

Appendix Figure S12
Ramirez et al.

Appendix Figure S12. ***Hormone-induce PR binding in 2D and 3D grown T47D cells***
Venn diagram of the 2D and 3D-exclusive PR binding sites (PRbs) from two replicates of ChIP-seq performed in 2D and 3D T47D cells in the presence and in the absence of 10 nM R5020 for 30 min. **B.** Progesterone (PR), estrogen (ER), and androgen receptor (AR) binding were examined in T47D cells cultured in monolayer (2D), in spheroids (3D), or in an ER+/PR+/AR+ patient-derived xenograft (PDX) model (Hickey et al., Nature Medicine 2021). Nuclear receptor binding in the tumor is recapitulated exclusively by 3D cells. **C.** HOMER de novo motif enrichment analysis for 3D-exclusive PR peaks.

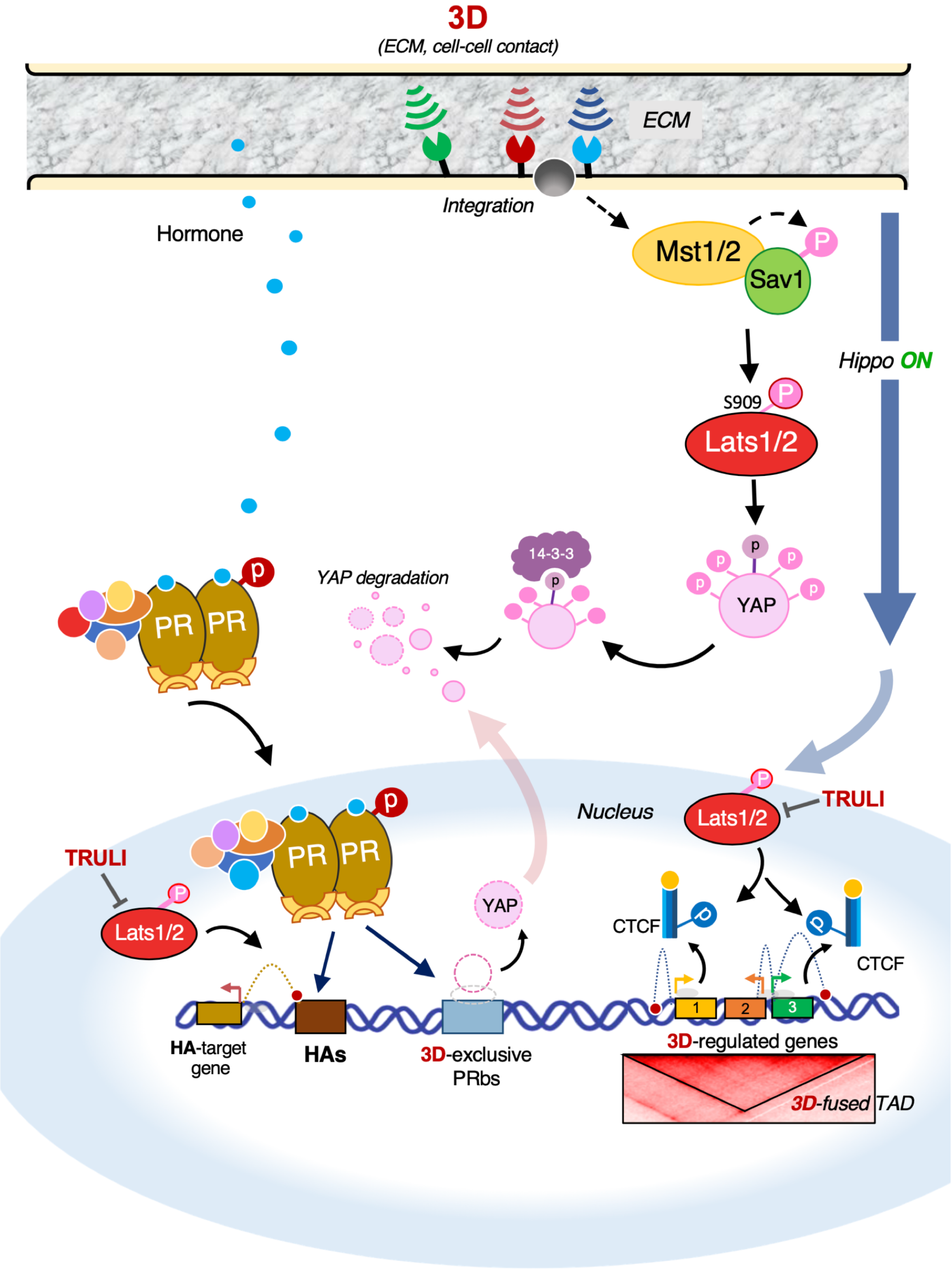

Appendix Figure S13. ***The Hippo kinase LATS1 controls CTCF binding and YAP nuclear availability in three-dimensionally grown breast cancer cells.*** T47D cells grown as spheroids are exposed to cell-cell contacts and to ECM. 3D-activated signals such as the Hippo pathway LATS kinase, impact on the cell nucleus in at least two ways: 1) the LATS1 kinase phosphorylates YAP promoting its cytoplasmic retention/degradation and 2) LATS1 also phosphorylates CTCF inducing its displacement from chromatin. The absence of these two proteins in the 3D nucleus determines the activity of a subset of genes specifically regulated in 3D condition and in turn, enhance the hormonal response.

Appendix Figure S13
Ramirez et al.

A

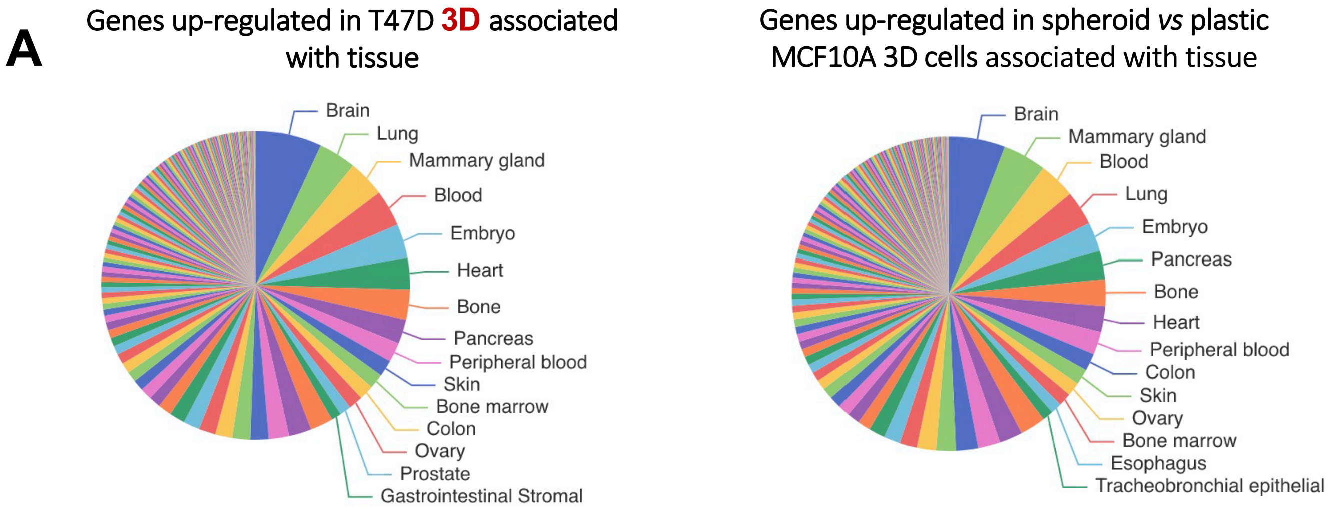

B

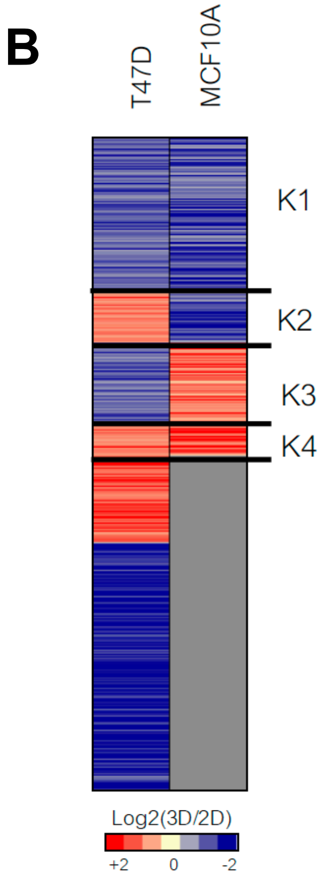

K1

ID	Source	Term ID	Term Name	p_{adj} (query_1)
10	GO:CC	GO:0022626	cytosolic ribosome	1.591×10^{-25}
3	GO:BP	GO:0002181	cytoplasmic translation	7.941×10^{-21}
4	GO:BP	GO:0009888	tissue development	8.780×10^{-14}
1	GO:MF	GO:0005515	protein binding	5.611×10^{-12}
2	GO:MF	GO:0005198	structural molecule activity	1.783×10^{-11}
11	GO:CC	GO:0030055	cell-substrate junction	1.988×10^{-9}
12	GO:CC	GO:0031982	vesicle	6.500×10^{-9}
5	GO:BP	GO:0008283	cell population proliferation	1.155×10^{-7}
6	GO:BP	GO:0040017	positive regulation of locomotion	2.029×10^{-7}
7	GO:BP	GO:0042274	ribosomal small subunit biogenesis	8.631×10^{-7}
13	GO:CC	GO:0120025	plasma membrane bounded cell projection	9.451×10^{-7}
14	GO:CC	GO:0032040	small-subunit processome	9.538×10^{-7}
15	GO:CC	GO:0005856	cytoskeleton	1.024×10^{-5}
16	GO:CC	GO:0016020	membrane	2.816×10^{-5}
17	GO:CC	GO:0062023	collagen-containing extracellular matrix	6.898×10^{-5}
8	GO:BP	GO:0007155	cell adhesion	1.454×10^{-4}
9	GO:BP	GO:1902531	regulation of intracellular signal transduction	1.926×10^{-4}

K2

ID	Source	Term ID	Term Name	p_{adj} (query_1)
1	GO:BP	GO:0048731	system development	2.022×10^{-8}
6	GO:BP	GO:0007399	nervous system development	2.578×10^{-7}
5	GO:CC	GO:0071944	cell periphery	2.256×10^{-4}
2	GO:BP	GO:0051171	regulation of nitrogen compound metabolic pro...	1.071×10^{-3}
7	GO:BP	GO:0045595	regulation of cell differentiation	1.476×10^{-3}
3	GO:BP	GO:0080090	regulation of primary metabolic process	3.694×10^{-3}
4	GO:BP	GO:0007155	cell adhesion	6.685×10^{-3}

K3

ID	Source	Term ID	Term Name	p_{adj} (query_1)
1	GO:MF	GO:0005515	protein binding	6.929×10^{-12}
3	GO:BP	GO:0007155	cell adhesion	1.488×10^{-11}
5	GO:CC	GO:0005925	focal adhesion	1.973×10^{-11}
6	GO:CC	GO:0005576	extracellular region	4.964×10^{-11}
7	GO:CC	GO:0071944	cell periphery	2.252×10^{-10}
4	GO:BP	GO:0006915	apoptotic process	9.914×10^{-8}
8	GO:CC	GO:0005930	axoneme	5.515×10^{-7}
2	GO:MF	GO:0098772	molecular function regulator activity	1.072×10^{-4}
9	GO:CC	GO:0009986	cell surface	7.725×10^{-4}

K4

ID	Source	Term ID	Term Name	p_{adj} (query_1)
1	GO:BP	GO:0007275	multicellular organism development	1.951×10^{-4}
5	GO:BP	GO:0032502	developmental process	4.224×10^{-4}
2	GO:BP	GO:0023051	regulation of signaling	4.783×10^{-4}
6	GO:BP	GO:0048856	anatomical structure development	5.011×10^{-4}
7	GO:BP	GO:0048732	gland development	1.706×10^{-3}
3	GO:BP	GO:0009719	response to endogenous stimulus	2.843×10^{-3}
4	GO:BP	GO:0007166	cell surface receptor signaling pathway	5.672×10^{-3}

Appendix Figure S14. ***Gene expression profiles of tumoral and non-tumoral mammary cells when shifted to a 3D environment***

A. Tissue association of up-regulated genes in T47D and MCF10A cells grown in 2D and 3D conditions. **B.** Gene clustering performed in T47D and MCF10A cells grown in 2D and 3D conditions is shown. *K2* and *K3* represent genes that are differentially expressed in tumoral *vs* non-tumoral model system once grown as spheres.

Appendix Figure S14
Ramirez et al.

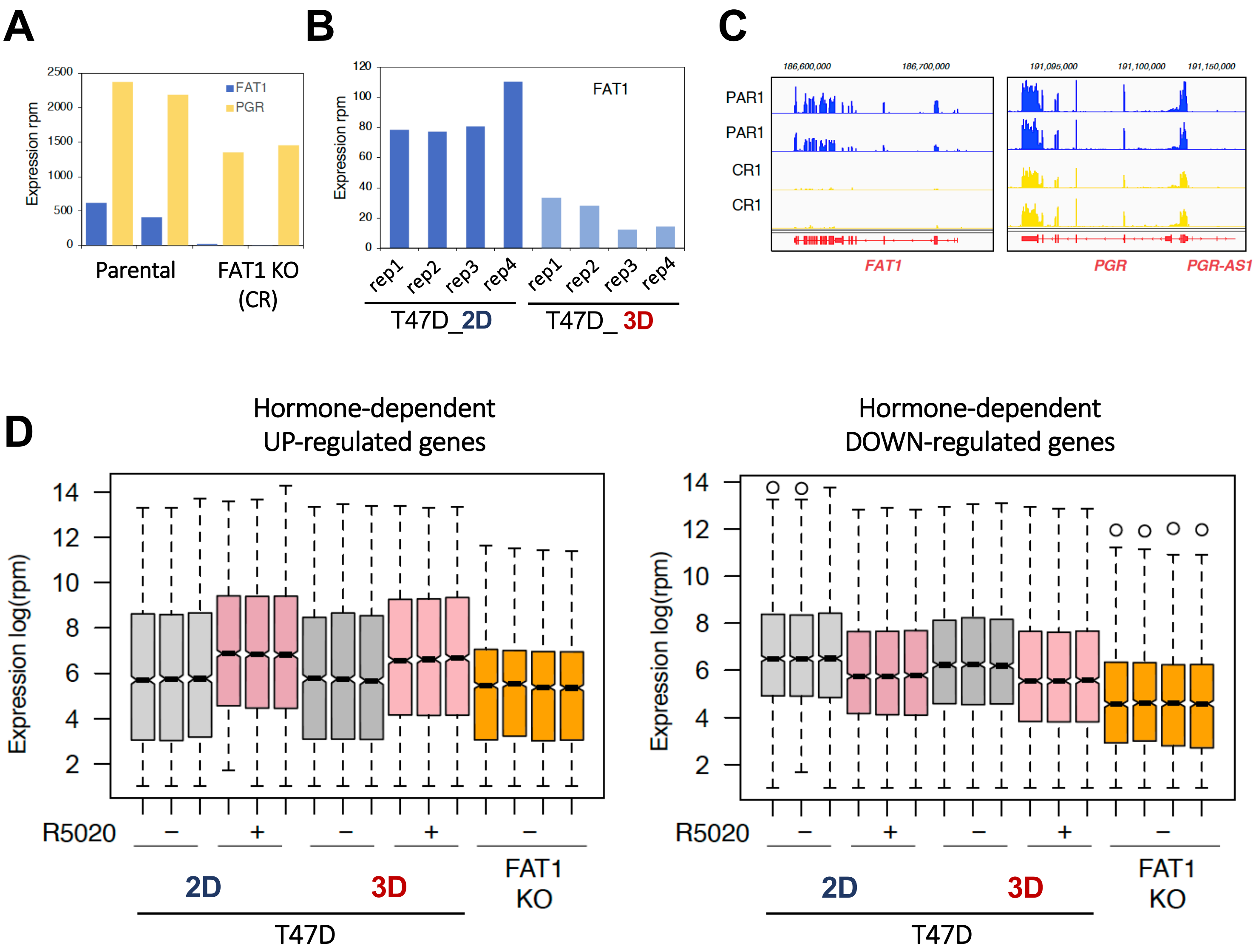

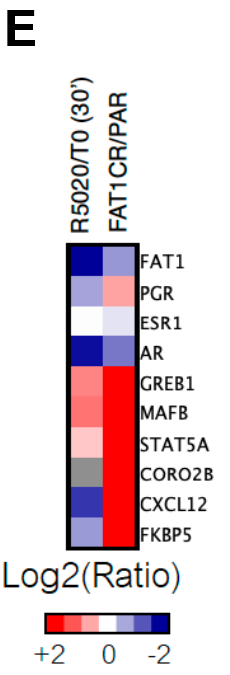

Appendix Figure S15. ***Connection between FAT1 KO and 3D-grown T47D cells***
A. *FAT1* and *PGR* levels obtained from Parental and FAT1KO cells is shown. RNA-seq data was obtained from Li et al., Cancer Cell 34, 893-905 e8 (2018) and analyzed as described in extended bioinformatics methods section. **B.** FAT1 mRNA levels in 2D and 3D T47D cells. **C.** Snapshot of the genome browser showing the FAT1 and PGR expression in Parental and FAT1KO cells. **D.** Hormone-dependent expression in 2D, 3D T47D and FAT1KO cells. Genes up- and down-regulated in the presence of R5020 are shown (left and right panels, respectively). The trend presented for the same genes in FAT1KO cells is also shown. **E.** List of genes differentially regulated by R5020 and FAT1.

Appendix Figure S15
Ramirez et al.

EXTENDED BIOINFORMATICS METHODS

ChIP-seq and ATAC-seq Peak Calling: Analysis of sequence data was carried out as previously described (2) with minor modifications. Reads were aligned to the hg38 human genome reference sequence (GRCh38) using Bowtie (3) and aligning parameters of uniqueness (-S –m1 –v2 –t –q). p values for the significance of ChIP-seq counts compared to input DNA were calculated as described (4) using a threshold of 10-8 and a false discovery rate (FDR) < 1%.

ChIP-seq Downstream Analysis

Average ChIP-seq signals of 50 bp windows around 1 kb upstream and downstream of annotated TSSs were calculated using the cis-regulatory annotation system (CEAS) (5).

ATAC-seq Downstream Analysis

PoissP wiggle generate from peaks calling (above) were used with Sitepro (5) to calculate the average nucleosome signal across genome coordinates of selected BED.

Motif analysis

Homer motif analysis (6) was conducted on all PRBs as represented in figure 2E). FIMO and SpaMo (3,5) were used respectively to calculate the frequency and the precise location of PR, FOXA1 and ERa corresponding binding motifs (HRE, FOXA1 and ERE). Matrixes used for each factor (PGR/MA0727.1) (ERS1/MA0112.2), (FOXA1/MA0148.2) are from Jaspar database (1).

RNA-seq

RNA extraction, RNA-seq library preparation, and qRT-PCR

RNA was isolated from cells cultured in 2D monolayer or as 3D spheroids with TRIzol reagent (Ambion), ethanol precipitated, and dissolved in sterile water. RNA concentration was measured with a Qubit fluorometer and RNA subjected to Bioanalyzer for quality control. Libraries were prepared using 1 µg of polyA+ RNA by PCR amplification of cDNA with bar-coded primers using the Illumina TruSeq kit at the CRG Genomic Facility. Libraries were sequenced using Illumina HIseq-2500 to obtain pair-ended (PE) 100-base-long reads.

For gene expression analysis, RNA (250 ng) was subjected to cDNA synthesis using the qScript cDNA Synthesis kit (Quanta Biosciences). qPCR was carried out using the LightCycler FastStart DNA Master SYBR Green I kit (Roche), and specific primers selected from the list of available designed primers at Primer Bank (https://pga.mgh.harvard.edu/primerbank) (7). As reference gene, GAPDH was used.

RNA-seq Pipeline and Differential Gene Expression Analysis

Sequencing adapters and low-quality ends were trimmed from the reads using Trimmomatic, using the parameters values recommended (8) and elsewhere (https://goo.gl/VzoqQq) (trimmomatic PE raw_fastq trimmed_fastq ILLUMINACLIP:TruSeq3-PE.fa:2:30:12:1:true LEADING:3 TRAILING:3 MAsXINFO:50:0.999 MINLEN:36). The trimmed reads were aligned to GRCh38 (9) using STAR (10).

First, the genome index files for STAR were generated with: star–runMode genomeGenerate–genomeDir GENOME_DIR–genomeFastaFiles genome_fasta–runThreadN slots–sjdbOverhang read_length–sjdbGTFfile sjdb–outFileNamePrefix GENOME_DIR/

Where genome_fasta is the FASTA file containing the GRCh38 sequence downloaded from the University of California Santa Cruz (UCSC) Genome Browser, excluding the random scaffolds and the alternative haplotypes; and sjdb is the GTF file with the GENCODE's V24 annotation.

Second, trimmed reads were aligned to the indexed genome with: star–genomeDir GENOME_DIR/–genomeLoad NoSharedMemory–runThreadN slots–outFilterType

"BySJout"–outFilterMultimapNmax 20–alignSJoverhangMin 8–alignSJDBoverhangMin 1–outFilterMismatchNmax 999–outFilterMismatchNoverLmax 0.04–alignIntronMin 20–alignIntronMax 1000000–alignMatesGapMax 1000000–readFilesIn read1 read2–outSAMtype BAM SortedByCoordinate–outTmpDir TMP_DIR/–outFileNamePrefix ODIR1/$sample_id.–outWigType bedGraph–readFilesCommand zcat.
Differences in gene expression were calculated by using a DESeq.R script for RNA analysis (available for download at Mendeley http://dx.doi.org/10.17632/mzjf96t3gc.5). Genes with fold change (FC) .} 1.5 (p value < 0.05; FDR < 0.01) were considered as significantly regulated. Sitepro profiles were generated with the script, provided in the CEAS package.

***In situ* Hi-C library preparation**
In situ Hi-C was performed as previously described (11) with the following modifications: (i) two million cells obtained from T47D cells cultured in 2D monolayer or as 3D spheroids were used as starting material; (ii) chromatin was initially digested with 100 U MboI (New England BioLabs) for 2 h, and then another 100 U (2 h incubation) and a final 100 U were added before overnight incubation; (iii) before fill-in with bio-dATP, nuclei were pelleted and resuspended in fresh 1 NEB2 buffer; (iv) ligation was performed overnight at 24°C with 10,000 cohesive end units per reaction; (v) de-cross-linked and purified DNA was sonicated to an average size of 300–400 bp with a Bioruptor Pico (Diagenode; seven cycles of 20 sec on and 60 sec off); (vi) DNA fragment-size selection was performed only after final library amplification; (vii) library preparation was performed with an NEBNext DNA Library Prep Kit (New England BioLabs) with 3 µl NEBNext adaptor in the ligation step; (viii) libraries were amplified for 8–12 cycles with Herculase II Fusion DNA Polymerase (Agilent) and were purified/size-selected with Agencourt AMPure XP beads (>200 bp). Hi-C library quality was assessed through ClaI digestion and low-coverage sequencing on an Illumina NextSeq500 instrument, after which every technical replicate (n=2) of each biological replicate (n=2) was sequenced at high coverage on an Illumina HiSeq2500 instrument. Data from technical replicates were pooled for downstream analysis. We sequenced >18 billion reads in total to obtain 0.78–1.21 billion valid interactions per time point per biological replicate.

***In situ* Hi-C data processing and normalization**
We processed Hi-C data by using an in-house pipeline based on TADbit (12). First, the quality of the reads was checked with FastQC to discard problematic samples and detect systematic artifacts. Trimmomatic (8) with the recommended parameters for paired-end reads was used to remove adaptor sequences and poor-quality reads (ILLUMINACLIP:TruSeq3-PE.fa:2:30:12:1:true; LEADING:3; TRAILING:3; MAsXINFO:targetLength:0.999; and MINLEN:36).
For mapping, a fragment-based strategy implemented in TADbit was used, which was similar to previously published protocols (13). Briefly, each side of the sequenced read was mapped in full length to the reference genome (GRCh38). After this step, if a read was not uniquely mapped, we assumed that the read was chimeric, owing to ligation of several DNA fragments. We next searched for ligation sites, discarding those reads in which no ligation site was found. The remaining reads were split as often as ligation sites were found.
Individual split read fragments were then mapped independently. These steps were repeated for each read in the input FASTQ files. Multiple fragments from a single uniquely mapped read resulted in a number of contacts identical to the number of possible pairs between the fragments. For example, if a single read was mapped through three fragments, a total of three contacts (all-versus-all) was represented in the final contact matrix. We used the TADbit filtering module to remove non-informative contacts and to create contact matrices. The different categories of filtered reads applied were:
1.Self-circle: reads coming from a single restriction enzyme (RE) fragment and pointing to the outside.

2.Dangling end: reads coming from a single RE fragment and pointing to the inside.
3.Error: reads coming from a single RE fragment and pointing in the same direction
4.Extra dangling end: reads coming from different RE fragments but that were sufficiently close and point to the inside; the distance threshold used was left to 500 bp (default), which was between percentiles 95 and 99 of average fragment lengths.
5. Duplicated: the combination of the start positions and directions of the reads was repeated, thus suggesting a PCR artifact; this filter removed only extra copies of the original pair.
6. Random breaks: the start position of one of the reads was too far from RE cutting site, possibly because of non-canonical enzymatic activity or random physical breaks; the threshold was set to 750 bp (default), >percentile 99.9.

From the resulting contact matrices, low-quality bins (those presenting low contact numbers) were removed, as implemented in TADbit's 'filter columns' routine. A single round of ICE normalization (14), also known as 'vanilla' normalization (16), was performed. That is, each cell in the Hi-C matrix was divided by the product of the interactions in its columns and the interactions in its row. Finally, all matrices were corrected to achieve an average content of one interaction per cell.

Identification of subnuclear compartments and topologically associated domains (TADs)

To segment the genome into A/B compartments, normalized Hi-C matrices at 100-kb resolution were corrected for decay as previously described, by grouping diagonals when the signal-to-noise ratio was below 0.05 (11). Corrected matrices were then split into chromosomal matrices and transformed into correlation matrices by using the Pearson product-moment correlation.

Normalized contacts matrices at 20-kb resolution were used to define TADs, and for visualization purposes, through a previously described method with default parameters (15,16). First, for each bin, an insulation index was obtained on the basis of the number of contacts between bins on each side of a given bin. Differences in the insulation index between both sides of the bin were computed, and borders were called, searching for minima within the insulation index. The insulation score of each border was determined as previously described (16), by using the difference in the delta vector between the local maximum to the left and the local minimum to the right of the boundary bin. This procedure resulted in a set of borders for each time point and replicate. To obtain a set of consensus borders along the time course, we proceeded in two steps: (i) merging borders of replicates and overlapping merged borders (that is, for each pair of replicates, we expanded the borders one bin on each side and kept only those borders present in both replicates as merged borders) and (ii) further expanding two extra bins (100 kb) on each side and determining the overlap to obtain a consensus set of borders common to any pair of time points.

Appendix Figure S3: Mutant or Translocated Estrogen Receptor Alpha (MOTERA) signature heatmap for our RNA-seq experiments of 3D versus 2D gene expression was generated recovering the log2 ratio (3Dvs2D) for each of the 24 genes belonging to the signature. Cluster 3.0 was used to generate the CDT file loaded on Java Tree View for heatmap visualization. Genes were ranked from the highest expressed to the lowest expressed.

EV1: Super-enhancers (SE) were calculated using Richard Young ROSE algorithm (https://bitbucket.org/young_computation/rose/src/master/) by applying H3K27ac data from ChIP-seq experiments in 2D and 3D conditions. Default parameter were used for SE computation. Gene association was done by using bedtools closest-features with the closest genes being the more highly expressed. A and B compartments were called using Homer (6) Sub-nuclear Compartment Analysis (PCA/Clustering).

EV3: Homer motif analysis (6) was conducted on 2D- and 3-D-specific ATAC-seq peaks using default parameters. Word clouds profile of transcription factor binding was generated using Toolkit analysis (http://dbtoolkit.cistrome.org/). The table reporting the GIGGLE score for each of the factor to be bound at these ATAC 2D and 3D peaks (retrieved from Toolkit) was used to extrapolate the frequency of the factor bound and plotted using text mining/word cloud using R package.

Appendix Figure S6: **A.** RAD21 ChIP-seq were analyzed as described in Material and Methods. For each ChIP-seq deepTools (Ramirez et al., 2014) was used to generate the meta-TAD analysis reporting the log10 of all RAD21 ChIP-seq normalized tags around and within the TAD fused. A random set of TADs was used as a control. Boxplots have been generate using data from panel A using R boxplot package. Two-way ANOVA test was used to calculate statistical significance.

Appendix Figure S8: **A.** *Drosophila M.* spike in CTCF ChIP-seq. Total tags form Drosophila M. retrieved in the ChIP experiment were counted and reported as a bar graph, for two biological replicates. Genome browser view of chromosome 2L form dm6 build was generated after H2av tags were compared with the input control for dm6. **B.** The IGV genome browser snapshot clear shows peaks of enrichment attesting the good immunoprecipitation of the H2av. For each CTCF sample we calculated the 3D vs 2D tag normalization factor given the spike-in tags retrieve in the H2av immunoprecipitation (as reported in the table). This factor was used to normalized the corresponding CTCF ChIP experiments (by applying this factor to the tag counts of the bam file). The resulted spike-in-normalized bam were used to downstream peak calling as described in material and methods. **C.** Heatmaps of Spike-in normalized CTCF ChIP-seq were generated using deepTools. From two biological replicates we generated one unique file by merging the two file and considering only the common peaks. We then used the 2D CTCF peaks to monitor the decrease of CTCF upon 3D growth in the presence or absence of TRULI (Panel C). We plotted heatmaps of enrichment using deepTools (Ramirez et al., 2014).

Appendix Figure S14: A. Comparison T47D vs MCF10A (Maguire et al., 2016). Gene up-regulated in T47D 3D and genes up-regulated in spheroid versus plastic MCF10A were analyzed for tissue-specific enrichment by employing SEdb2.0 (https://bio.liclab.net/sedb/analysis_gene.php). **B.** Heatmap of T47D 3D growth-regulated genes, compared to MCF10A spheroid versus plastic. Only 50% of the total T47D was detectable in MFC10A with various degree of expression inversions as in cluster (K) 2 and K3. Clusters K1 and K4 show parallel gene expression for both up- and down-regulation between the two cell types. Table of Gene Ontology (GO) for the cluster reported in the heatmap.

Appendix Figure S15: **A.** Expression levels of FAT1 and PGR in parental and FAT1 KO (CR) cells (Li et al., 2018). **B.** Expression of FAT1 in our T47D cells both growth in 2D and 3D. For both conditions the four replicate experiments levels are reported. **C.** Genome browser view of FAT1 and PGR gene expression levels. **D.** Boxplot comparison of hormone-dependent up- and down-regulated genes in in both 2D and 3D with and without R5020 treatment. The expression levels of these groups are compared also with the same genes but in the RNA-seq from FAT1 KO cells (Li et al., 2018). **E.** Heatmap of expression of important hormone regulated genes.

All sequenced data have been deposited in the GEO database under the accession number: **GSE247777**.

Appendix references

1. Sandelin, A., Alkema, W., Engstrom, P., Wasserman, W.W. and Lenhard, B. (2004) JASPAR: an open-access database for eukaryotic transcription factor binding profiles. Nucleic Acids Res, 32, D91-94.

2. Ferrari, R., de Llobet Cucalon, L.I., Di Vona, C., Le Dilly, F., Vidal, E., Lioutas, A., Oliete, J.Q., Jochem, L., Cutts, E., Dieci, G. et al. (2020) TFIIIC Binding to Alu Elements Controls Gene Expression via Chromatin Looping and Histone Acetylation. Mol Cell, 77, 475-487 e411.

3. Langmead, B., Trapnell, C., Pop, M. and Salzberg, S.L. (2009) Ultrafast and memory-efficient alignment of short DNA sequences to the human genome. Genome Biol, 10, R25.

4. Pellegrini, M. and Ferrari, R. (2012) Epigenetic analysis: ChIP-chip and ChIPseq. Methods Mol Biol, 802, 377-387.

5. Shin, H., Liu, T., Manrai, A.K. and Liu, X.S. (2009) CEAS: cis-regulatory element annotation system. Bioinformatics, 25, 2605-2606.

6. Heinz, S., Benner, C., Spann, N., Bertolino, E., Lin, Y.C., Laslo, P., Cheng, J.X., Murre, C., Singh, H. and Glass, C.K. (2010) Simple combinations of lineagedetermining transcription factors prime cis-regulatory elements required for macrophage and B cell identities. Mol Cell, 38, 576-589.

7. Wang, X., Spandidos, A., Wang, H. and Seed, B. (2012) PrimerBank: a PCR primer database for quantitative gene expression analysis, 2012 update. Nucleic Acids Res, 40, D1144-1149.

8. Bolger, A.M., Lohse, M. and Usadel, B. (2014) Trimmomatic: a flexible trimmer for Illumina sequence data. Bioinformatics, 30, 2114-2120.

9. Lander, E.S., Linton, L.M., Birren, B., Nusbaum, C., Zody, M.C., Baldwin, J., Devon, K., Dewar, K., Doyle, M., FitzHugh, W. et al. (2001) Initial sequencing and analysis of the human genome. Nature, 409, 860-921."

10. Dobin, A., Davis, C.A., Schlesinger, F., Drenkow, J., Zaleski, C., Jha, S., Batut, P., Chaisson, M. and Gingeras, T.R. (2013) STAR: ultrafast universal RNA-seq aligner. Bioinformatics, 29, 15-21.

11. Rao, S.S., Huntley, M.H., Durand, N.C., Stamenova, E.K., Bochkov, I.D., Robinson, J.T., Sanborn, A.L., Machol, I., Omer, A.D., Lander, E.S. et al. (2014) A 3D map of the human genome at kilobase resolution reveals principles of chromatin looping. Cell, 159, 1665-1680.

12. Serra, F., Bau, D., Goodstadt, M., Castillo, D., Filion, G.J. and Marti-Renom, M.A. (2017) Automatic analysis and 3D-modelling of Hi-C data using TADbit reveals structural features of the fly chromatin colors. PLoS Comput Biol, 13, e1005665.

13. Ay, F., Vu, T.H., Zeitz, M.J., Varoquaux, N., Carette, J.E., Vert, J.P., Hoffman, A.R. and Noble, W.S. (2015) Identifying multi-locus chromatin contacts in human cells using tethered multiple 3C. BMC Genomics, 16, 121.

14. Imakaev, M., Fudenberg, G., McCord, R.P., Naumova, N., Goloborodko, A., Lajoie, B.R., Dekker, J. and Mirny, L.A. (2012) Iterative correction of Hi-C data reveals hallmarks of chromosome organization. Nature methods, 9, 999-1003.

15. Giorgetti, L., Lajoie, B.R., Carter, A.C., Attia, M., Zhan, Y., Xu, J., Chen, C.J., Kaplan, N., Chang, H.Y., Heard, E. et al. (2016) Structural organization of the inactive X chromosome in the mouse. Nature, 535, 575-579.

16. Crane, E., Bian, Q., McCord, R.P., Lajoie, B.R., Wheeler, B.S., Ralston, E.J., Uzawa, S., Dekker, J. and Meyer, B.J. (2015) Condensin-driven remodelling of X chromosome topology during dosage compensation. Nature, 523, 240-244.

17. Ramírez F, Dündar F, Diehl S, Grüning BA, Manke T. deepTools: a flexible platform for exploring deep-sequencing data. Nucleic Acids Res. 2014 Jul;42(Web Server issue):W187-91. doi: 10.1093/nar/gku365.
