# Supplementary figures and images for: LATS1 controls CTCF chromatin occupancy and hormonal response of 3D-grown breast cancer cells
Source: EMBO J. 2024 Apr 2;43(9):5. doi: 10.1038/s44318-024-00080-x (PMC11066098; doi:10.1038/s44318-024-00080-x)

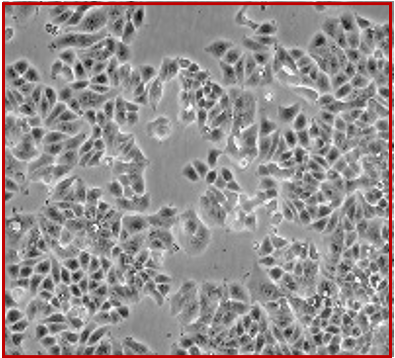

Supplement: Supplementary file 2 — Source data Fig. 1 [file 44318_2024_80_MOESM2_ESM.zip › Figura 1/A/Image_T47D_2D.png]

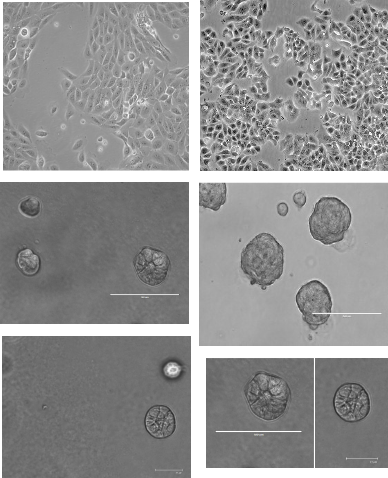

Supplement: Supplementary file 2 — Source data Fig. 1 [file 44318_2024_80_MOESM2_ESM.zip › Figura 1/A/1A.tif]

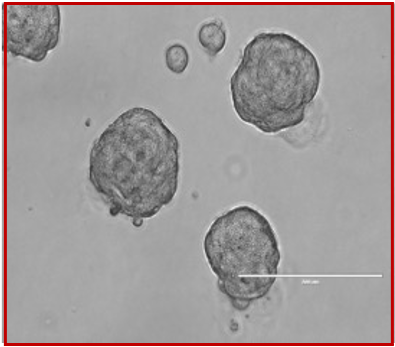

Supplement: Supplementary file 2 — Source data Fig. 1 [file 44318_2024_80_MOESM2_ESM.zip › Figura 1/A/Imagen_T47D_3D.png]

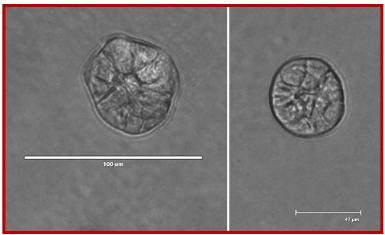

Supplement: Supplementary file 2 — Source data Fig. 1 [file 44318_2024_80_MOESM2_ESM.zip › Figura 1/A/Imagen_MCF10A_3D_merge.png]

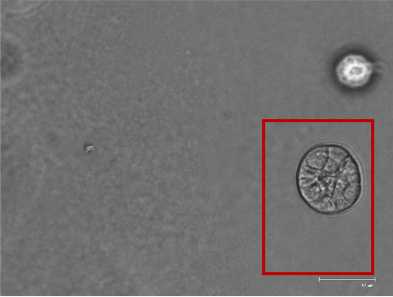

Supplement: Supplementary file 2 — Source data Fig. 1 [file 44318_2024_80_MOESM2_ESM.zip › Figura 1/A/Imagen_MCF10A_3D_B.png]

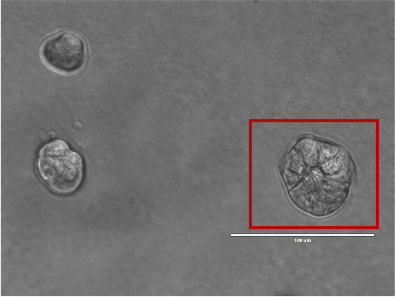

Supplement: Supplementary file 2 — Source data Fig. 1 [file 44318_2024_80_MOESM2_ESM.zip › Figura 1/A/Image_MCF10A_3D_A.png]

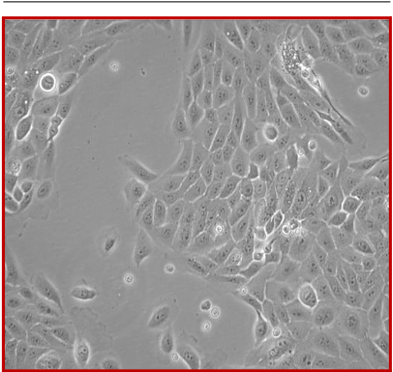

Supplement: Supplementary file 2 — Source data Fig. 1 [file 44318_2024_80_MOESM2_ESM.zip › Figura 1/A/Image_MCF10A_2D.png]

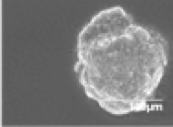

Supplement: Supplementary file 2 — Source data Fig. 1 [file 44318_2024_80_MOESM2_ESM.zip › Figura 1/C/10 days.tif]

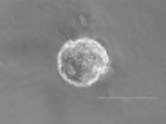

Supplement: Supplementary file 2 — Source data Fig. 1 [file 44318_2024_80_MOESM2_ESM.zip › Figura 1/C/8 days.tif]

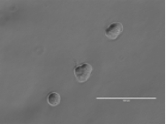

Supplement: Supplementary file 2 — Source data Fig. 1 [file 44318_2024_80_MOESM2_ESM.zip › Figura 1/C/2 days.tif]

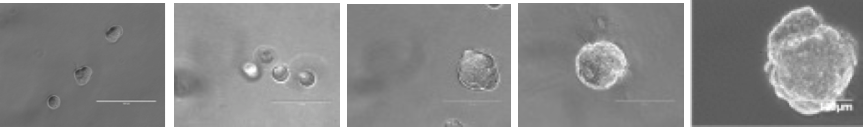

Supplement: Supplementary file 2 — Source data Fig. 1 [file 44318_2024_80_MOESM2_ESM.zip › Figura 1/C/1C.tif]

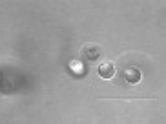

Supplement: Supplementary file 2 — Source data Fig. 1 [file 44318_2024_80_MOESM2_ESM.zip › Figura 1/C/4 days.tif]

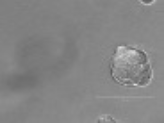

Supplement: Supplementary file 2 — Source data Fig. 1 [file 44318_2024_80_MOESM2_ESM.zip › Figura 1/C/6 days.tif]

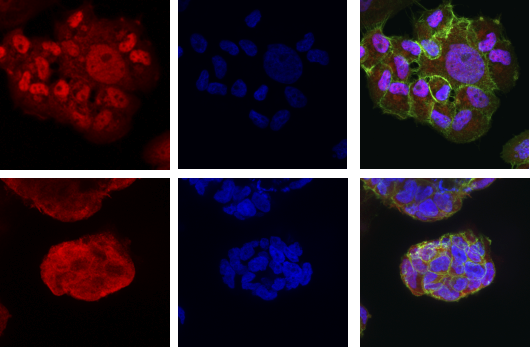

Supplement: Supplementary file 2 — Source data Fig. 1 [file 44318_2024_80_MOESM2_ESM.zip › Figura 1/D/1D.tif]

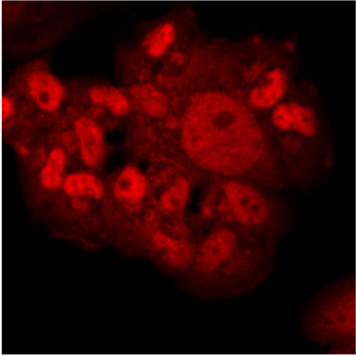

Supplement: Supplementary file 2 — Source data Fig. 1 [file 44318_2024_80_MOESM2_ESM.zip › Figura 1/D/Image_2D_YAP.png]

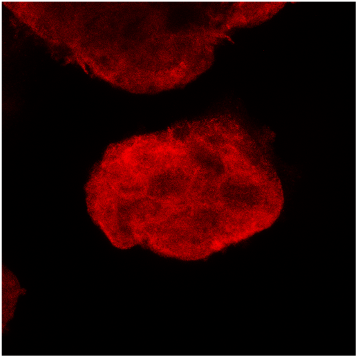

Supplement: Supplementary file 2 — Source data Fig. 1 [file 44318_2024_80_MOESM2_ESM.zip › Figura 1/D/Image_3D_YAP.png]

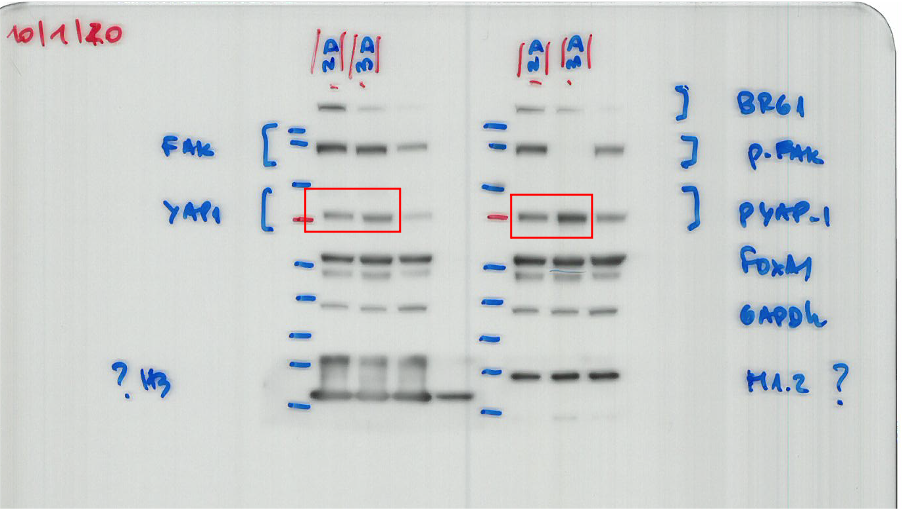

Supplement: Supplementary file 2 — Source data Fig. 1 [file 44318_2024_80_MOESM2_ESM.zip › Figura 1/D/Western_YAP_pYAP.png]

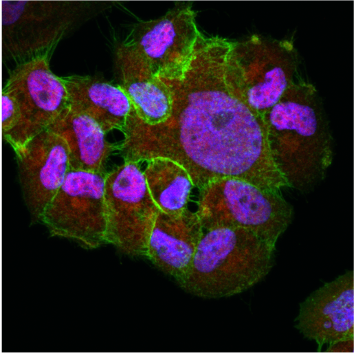

Supplement: Supplementary file 2 — Source data Fig. 1 [file 44318_2024_80_MOESM2_ESM.zip › Figura 1/D/Image_2D_merge.png]

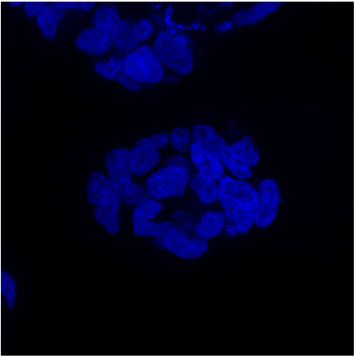

Supplement: Supplementary file 2 — Source data Fig. 1 [file 44318_2024_80_MOESM2_ESM.zip › Figura 1/D/Image_3D_DAPI.png]

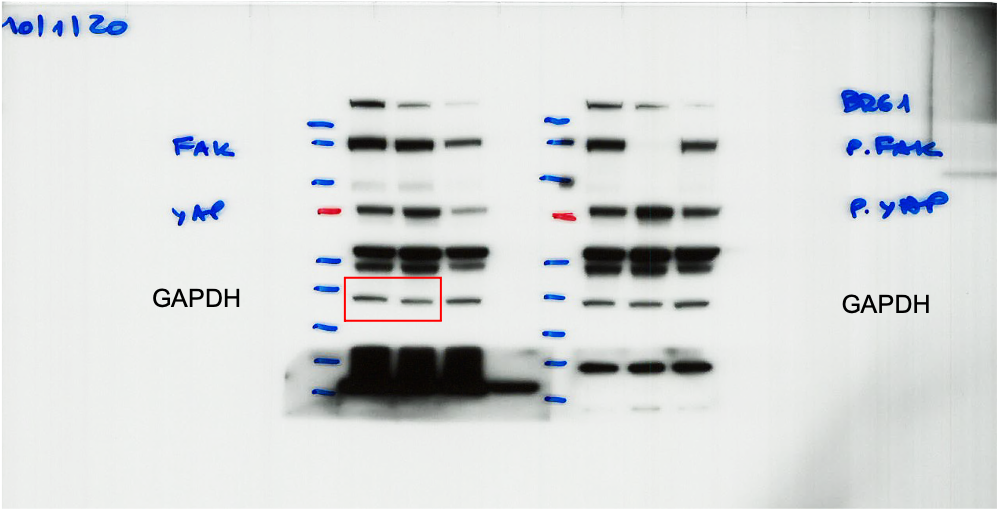

Supplement: Supplementary file 2 — Source data Fig. 1 [file 44318_2024_80_MOESM2_ESM.zip › Figura 1/D/Western_GAPDH.png]

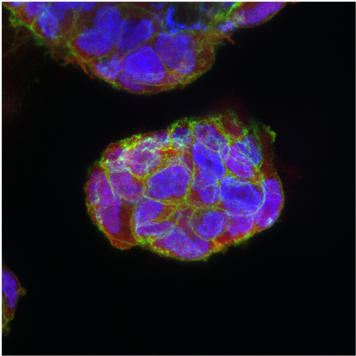

Supplement: Supplementary file 2 — Source data Fig. 1 [file 44318_2024_80_MOESM2_ESM.zip › Figura 1/D/Image_3D_merge.png]

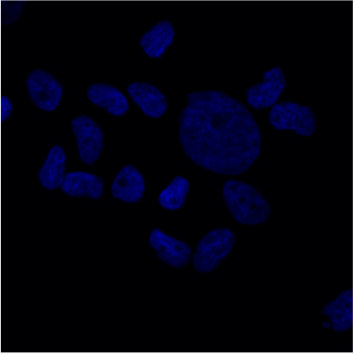

Supplement: Supplementary file 2 — Source data Fig. 1 [file 44318_2024_80_MOESM2_ESM.zip › Figura 1/D/Image_2D_DAPI.png]

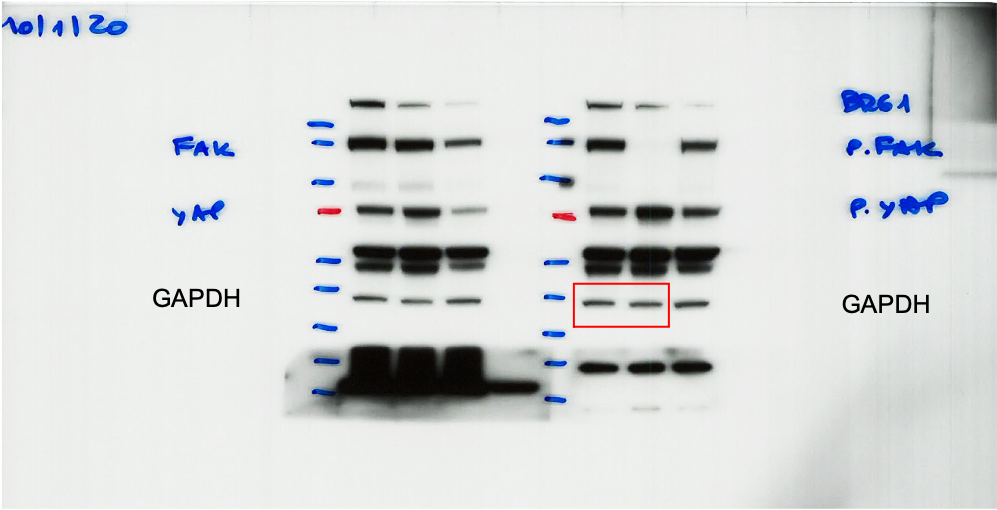

Supplement: Supplementary file 2 — Source data Fig. 1 [file 44318_2024_80_MOESM2_ESM.zip › Figura 1/E/Western_GAPDH.png]

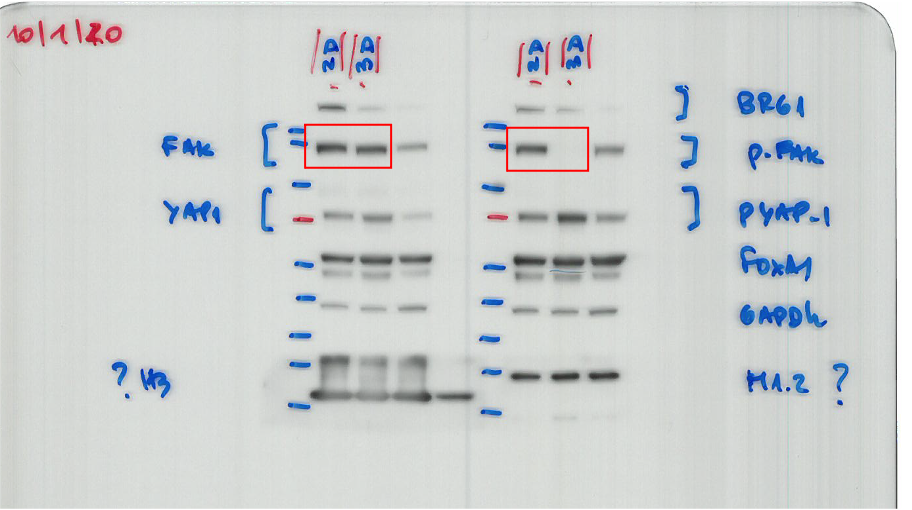

Supplement: Supplementary file 2 — Source data Fig. 1 [file 44318_2024_80_MOESM2_ESM.zip › Figura 1/E/Western_FAK_pFAK.png]

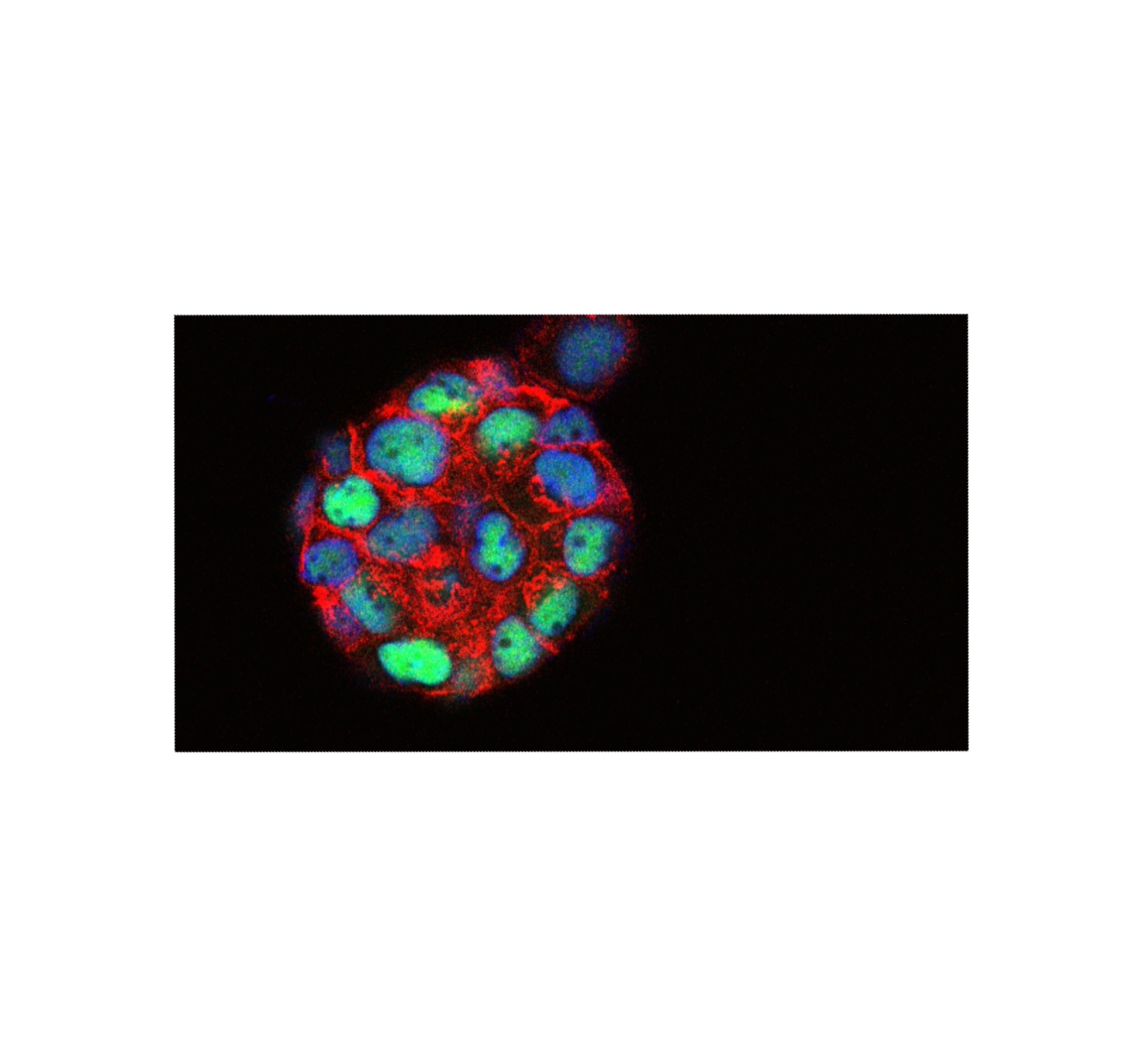

Supplement: Supplementary file 2 — Source data Fig. 1 [file 44318_2024_80_MOESM2_ESM.zip › Figura 1/B/Image_3D_PR_Bcatenin_DAPI_B.png]

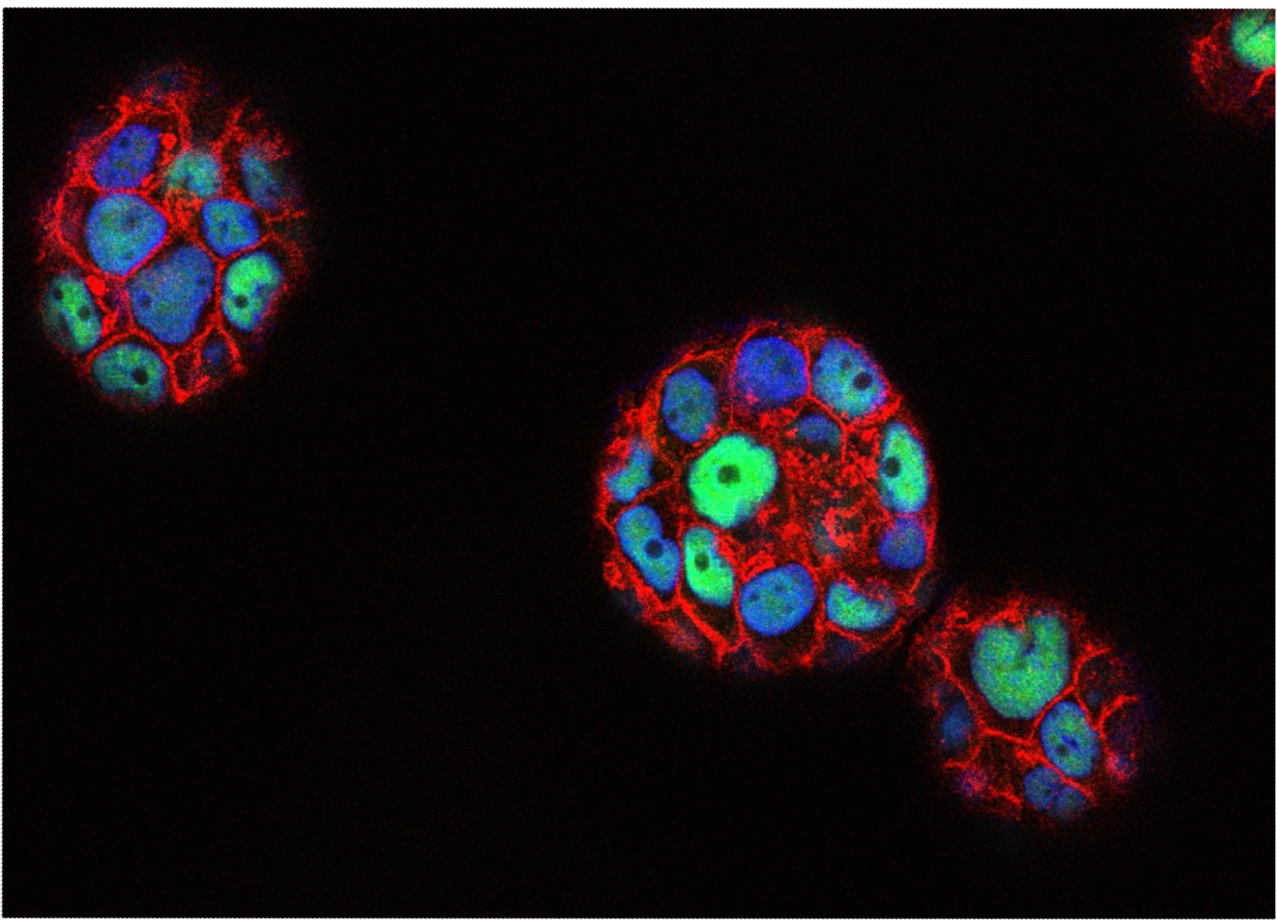

Supplement: Supplementary file 2 — Source data Fig. 1 [file 44318_2024_80_MOESM2_ESM.zip › Figura 1/B/Image_3D_PR_Bcatenin_CAPI_A.png]

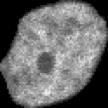

Supplement: Supplementary file 3 — Source data Fig. 2 [file 44318_2024_80_MOESM3_ESM.zip › Figura 2/A/2A_1.tif]

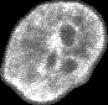

Supplement: Supplementary file 3 — Source data Fig. 2 [file 44318_2024_80_MOESM3_ESM.zip › Figura 2/A/2A_3.tif]

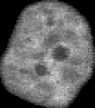

Supplement: Supplementary file 3 — Source data Fig. 2 [file 44318_2024_80_MOESM3_ESM.zip › Figura 2/A/2A_2.tif]

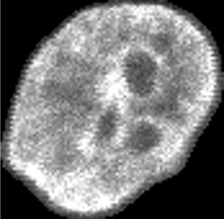

Supplement: Supplementary file 3 — Source data Fig. 2 [file 44318_2024_80_MOESM3_ESM.zip › Figura 2/A/Image_3D_A.png]

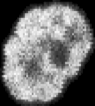

Supplement: Supplementary file 3 — Source data Fig. 2 [file 44318_2024_80_MOESM3_ESM.zip › Figura 2/A/2A_4.tif]

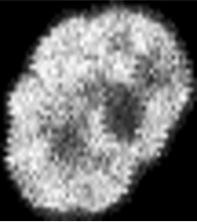

Supplement: Supplementary file 3 — Source data Fig. 2 [file 44318_2024_80_MOESM3_ESM.zip › Figura 2/A/Imagen_3D_B.png]

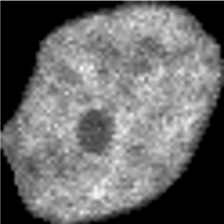

Supplement: Supplementary file 3 — Source data Fig. 2 [file 44318_2024_80_MOESM3_ESM.zip › Figura 2/A/Image_2D_A.png]

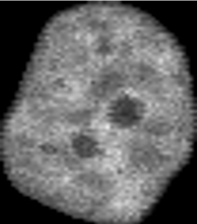

Supplement: Supplementary file 3 — Source data Fig. 2 [file 44318_2024_80_MOESM3_ESM.zip › Figura 2/A/Image_2D_B.png]

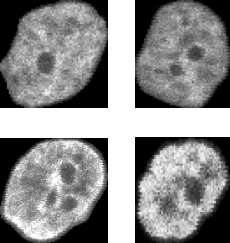

Supplement: Supplementary file 3 — Source data Fig. 2 [file 44318_2024_80_MOESM3_ESM.zip › Figura 2/A/2A.tif]

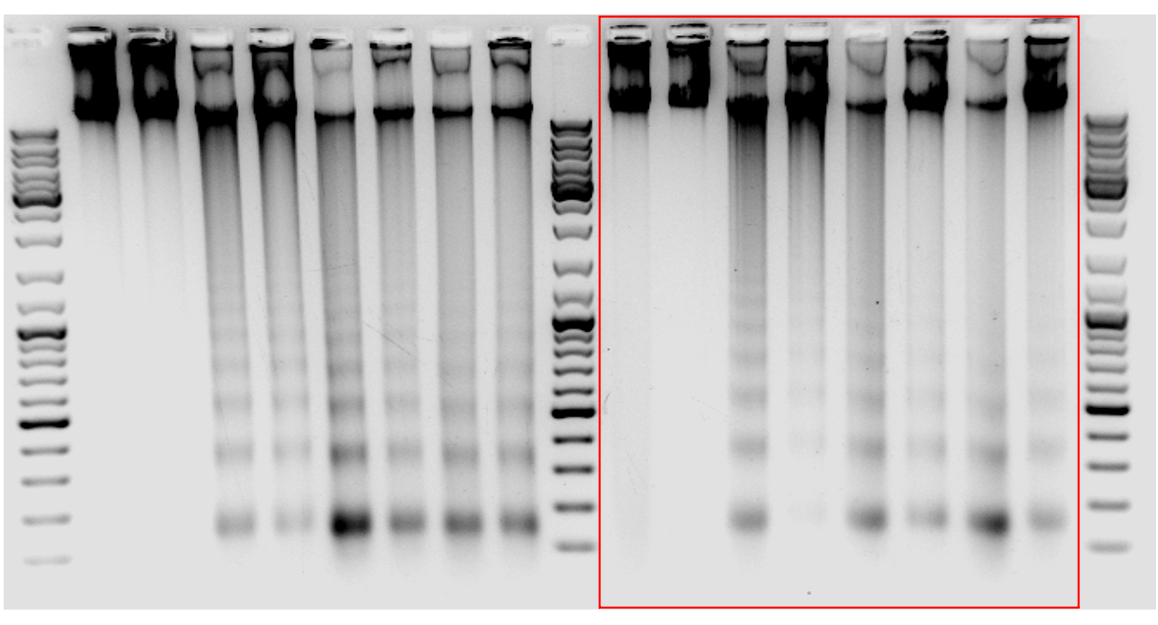

Supplement: Supplementary file 3 — Source data Fig. 2 [file 44318_2024_80_MOESM3_ESM.zip › Figura 2/B/Agarose_gel_MNase_2D_3D.png]

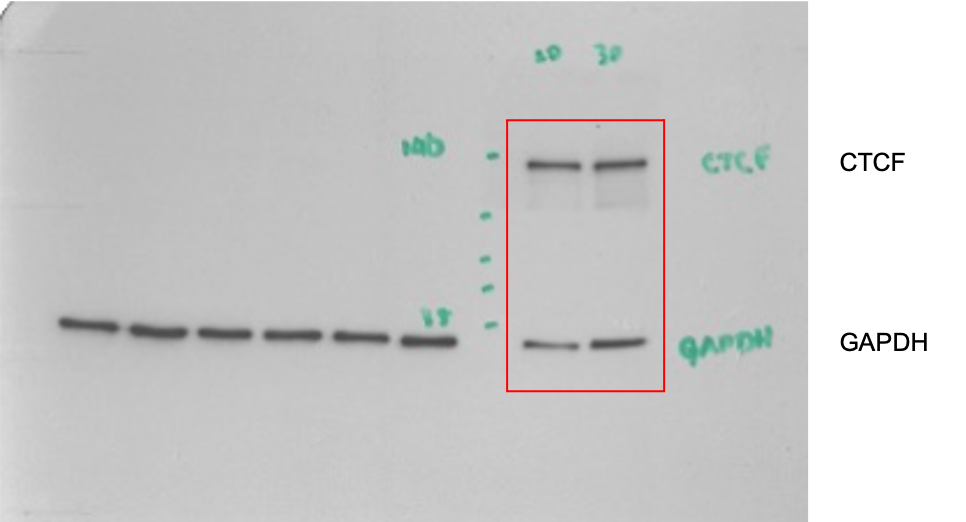

Supplement: Supplementary file 4 — Source data Fig. 4 [file 44318_2024_80_MOESM4_ESM.zip › Figura 4/A/Western_CTCF_2D_3D.png]

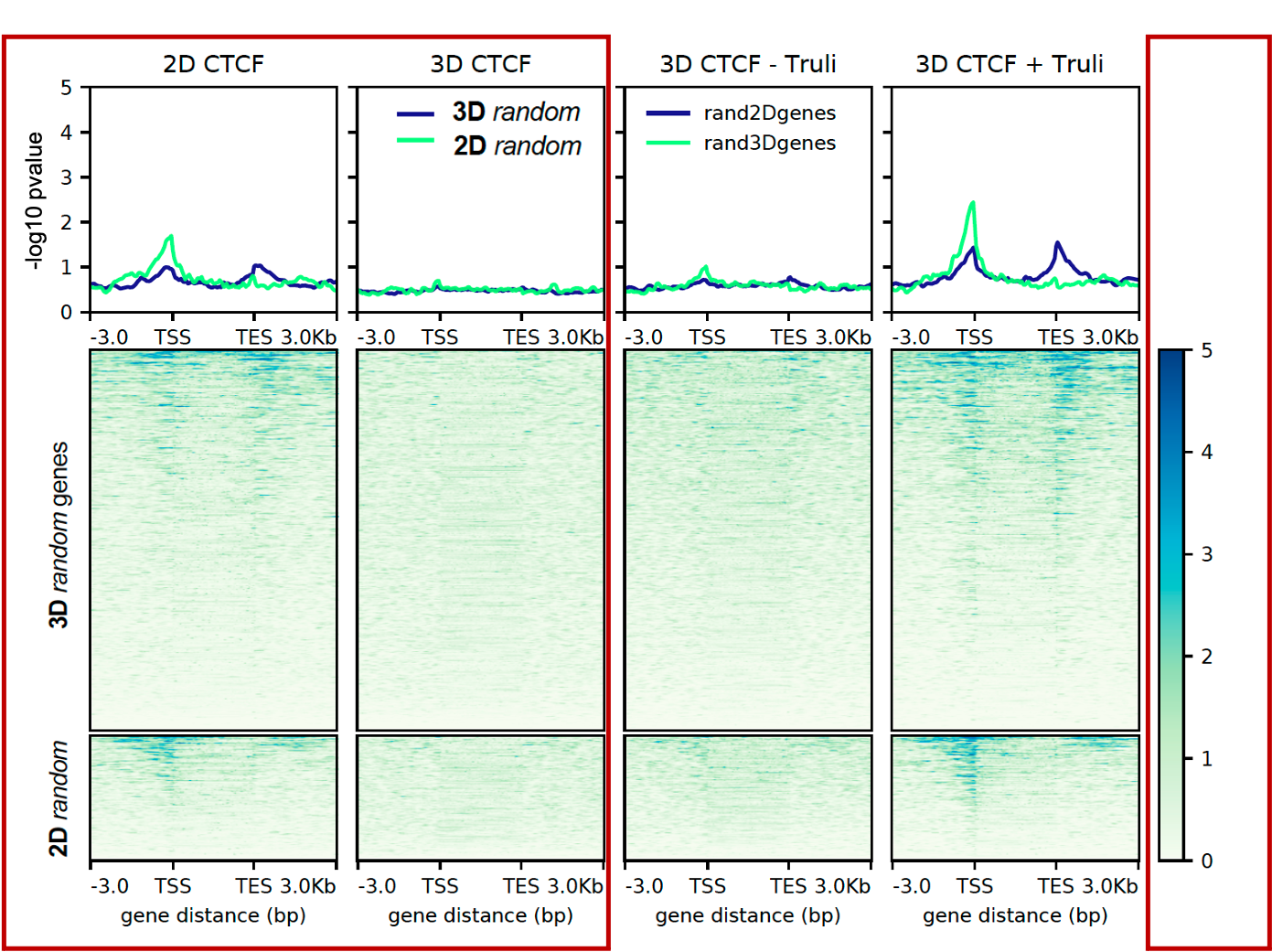

Supplement: Supplementary file 4 — Source data Fig. 4 [file 44318_2024_80_MOESM4_ESM.zip › Figura 4/D/Image_CTCF_2D_3D_random genes.png]

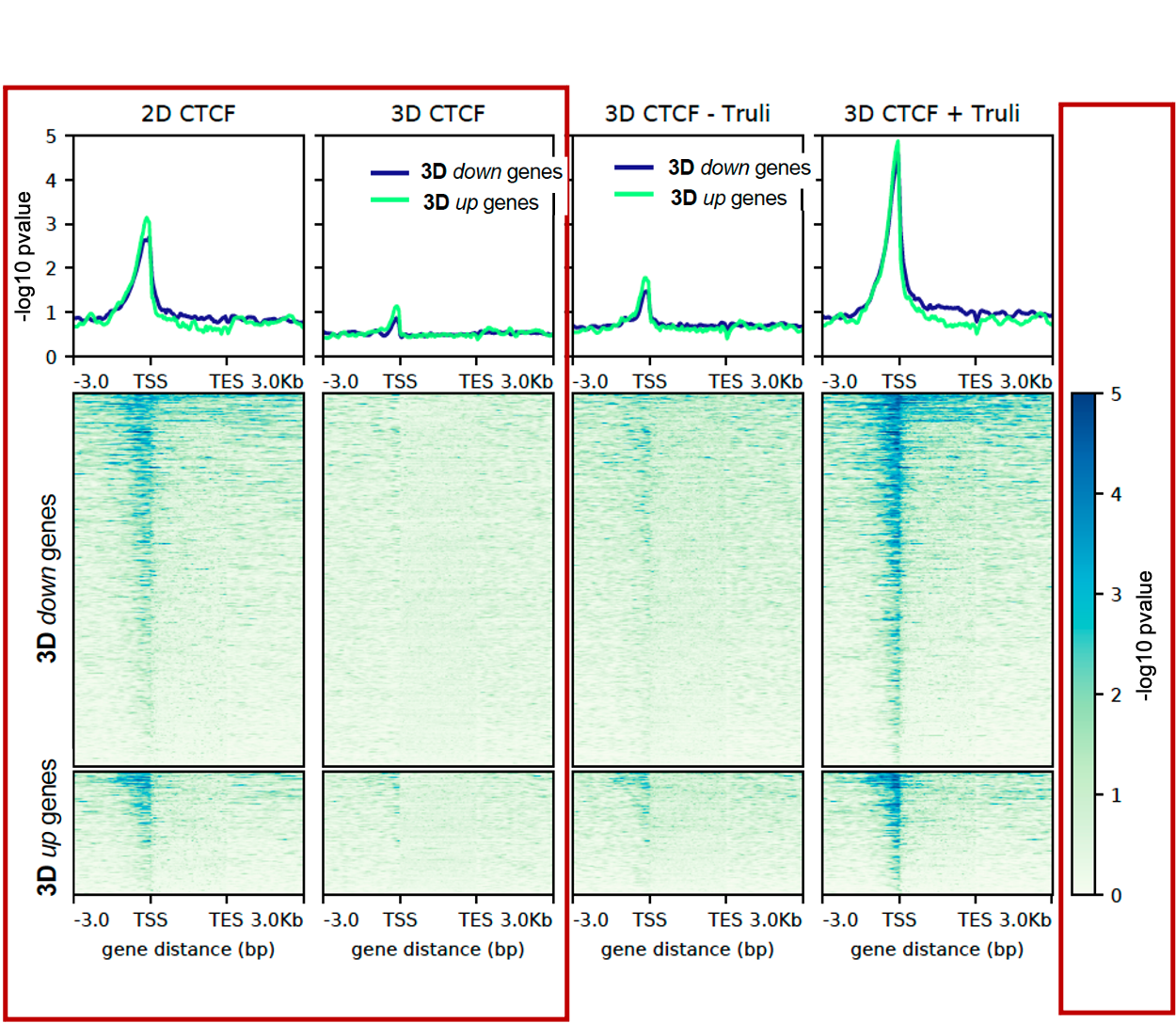

Supplement: Supplementary file 4 — Source data Fig. 4 [file 44318_2024_80_MOESM4_ESM.zip › Figura 4/D/Image_CTCF_2D_3D_Up_Down_genes.png]

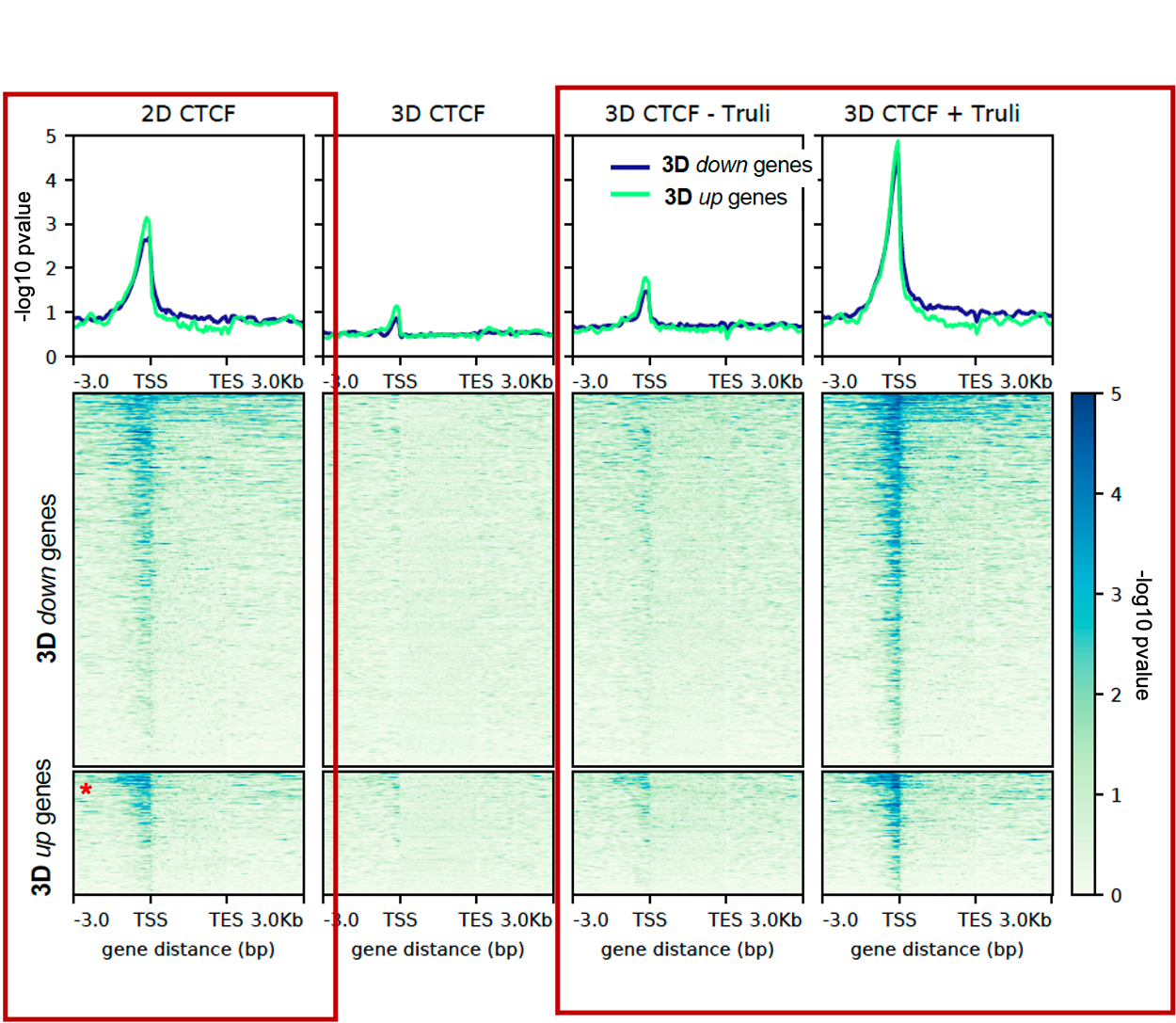

Supplement: Supplementary file 4 — Source data Fig. 4 [file 44318_2024_80_MOESM4_ESM.zip › Figura 4/E/Image_CTCF_2D_3D_truli_up_down_genes.png]

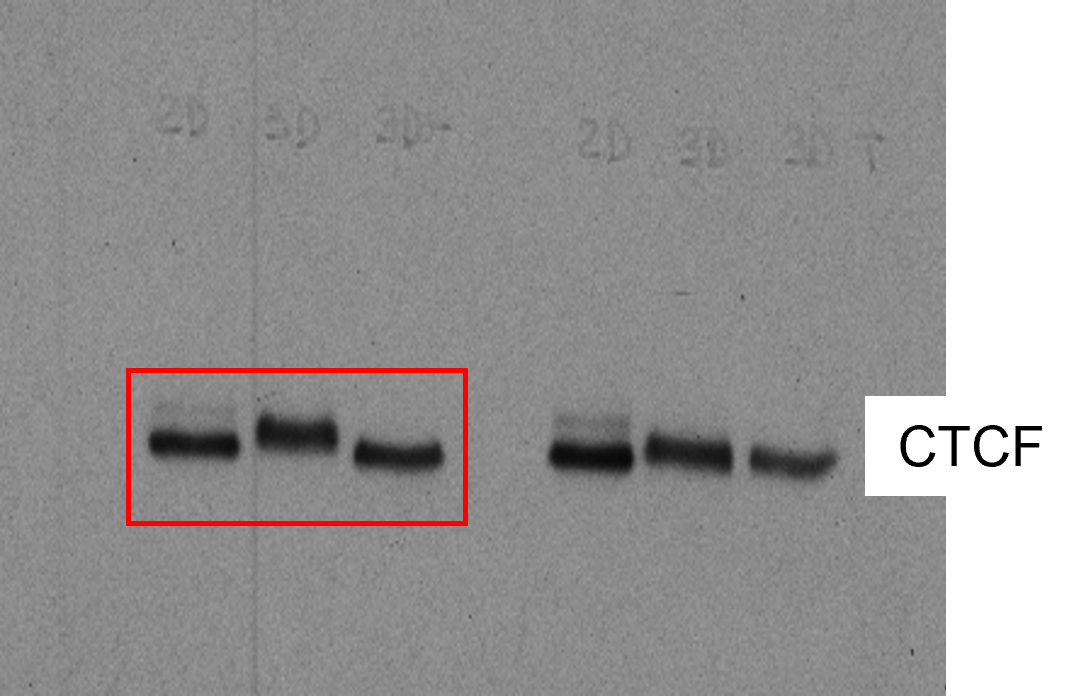

Supplement: Supplementary file 5 — Source data Fig. 5 [file 44318_2024_80_MOESM5_ESM.zip › Figure 5/5A/western_CTCF.png]

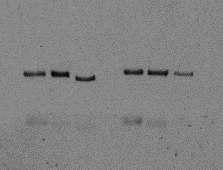

Supplement: Supplementary file 5 — Source data Fig. 5 [file 44318_2024_80_MOESM5_ESM.zip › Figure 5/5A/western_BRG1.png]

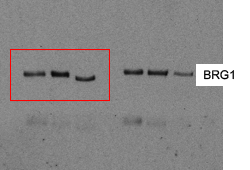

Supplement: Supplementary file 5 — Source data Fig. 5 [file 44318_2024_80_MOESM5_ESM.zip › Figure 5/5A/5A_BRG1.tif]

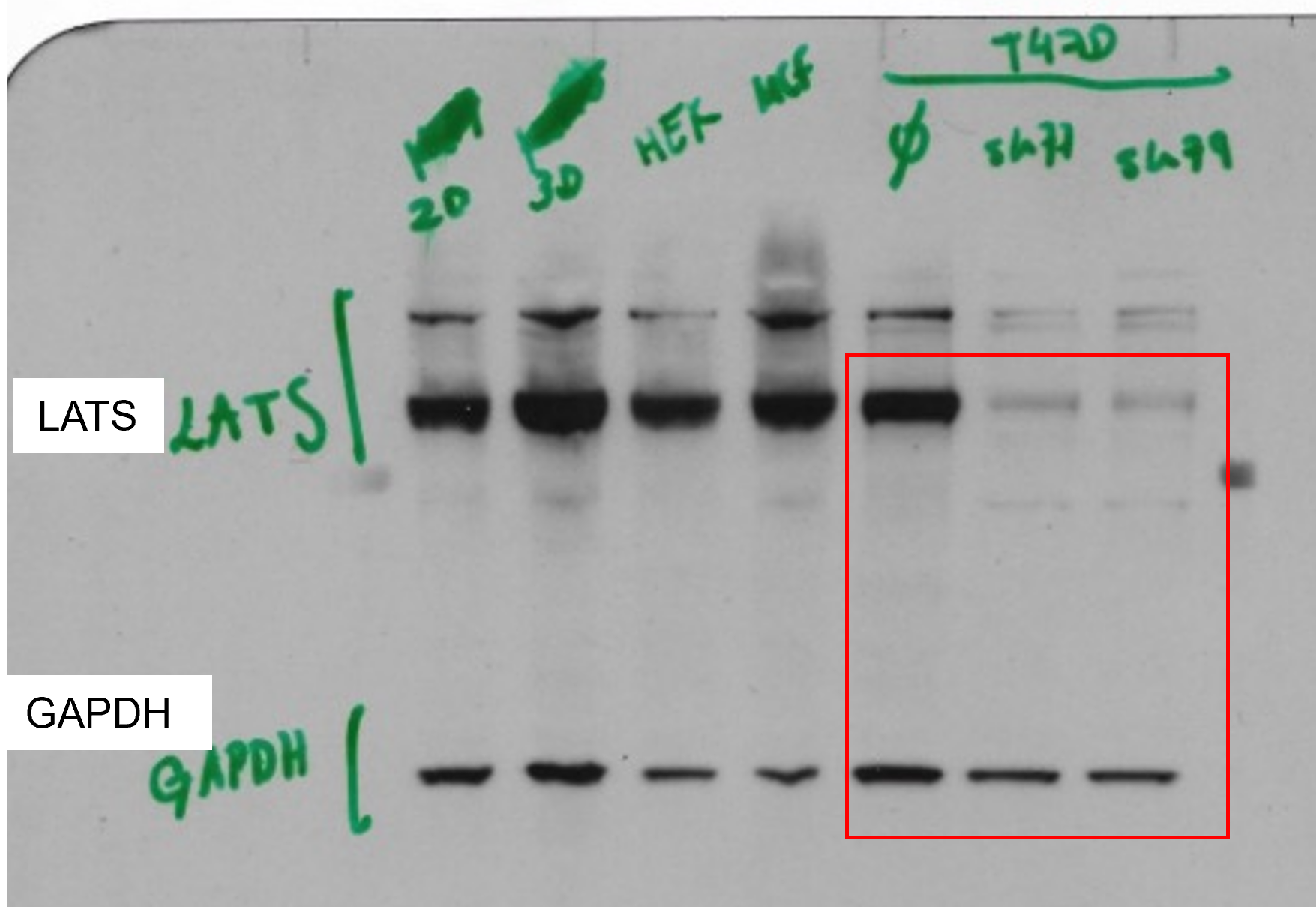

Supplement: Supplementary file 5 — Source data Fig. 5 [file 44318_2024_80_MOESM5_ESM.zip › Figure 5/5C/western_LATS_GAPDH.png]

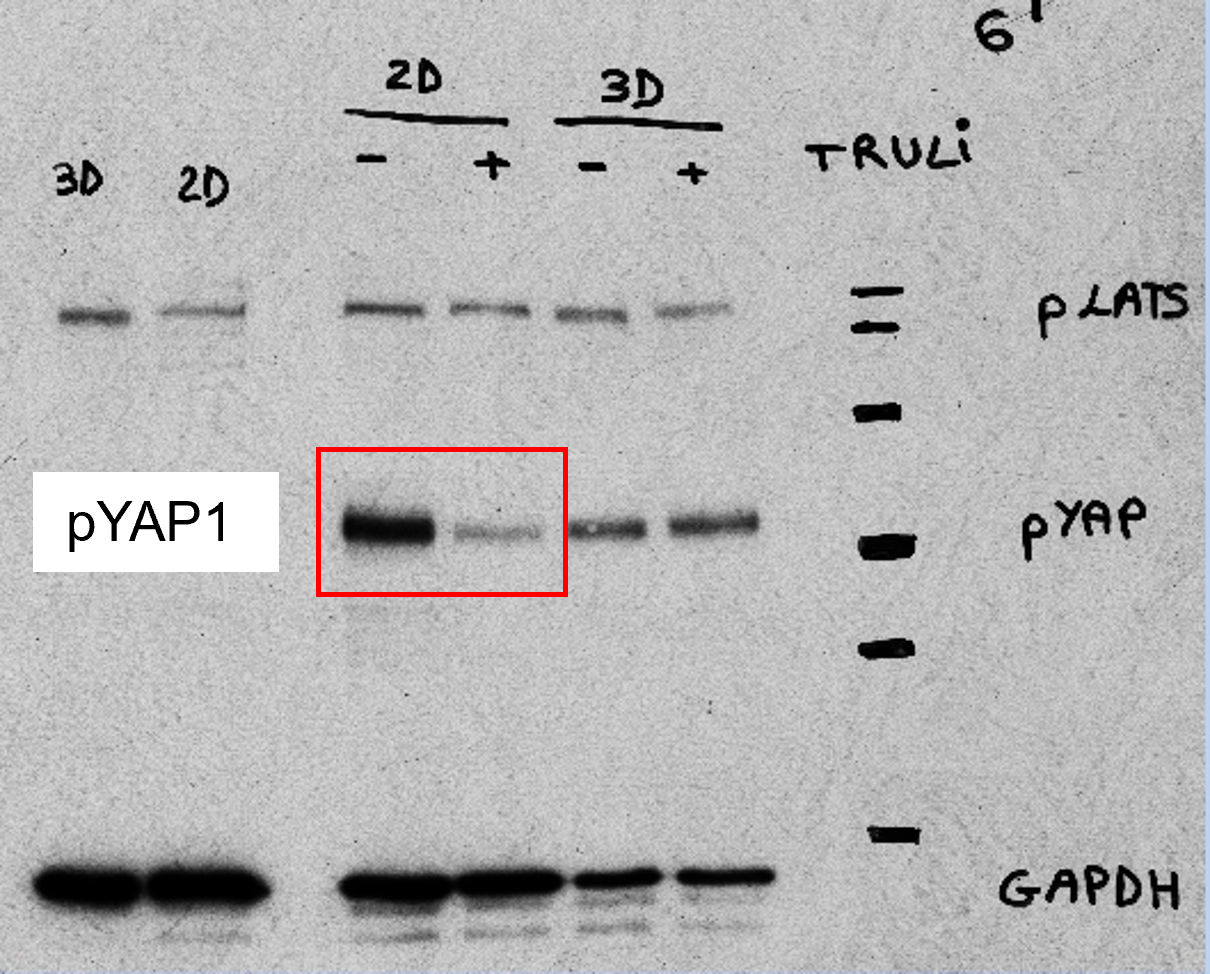

Supplement: Supplementary file 5 — Source data Fig. 5 [file 44318_2024_80_MOESM5_ESM.zip › Figure 5/5B/western_pYAP1.png]

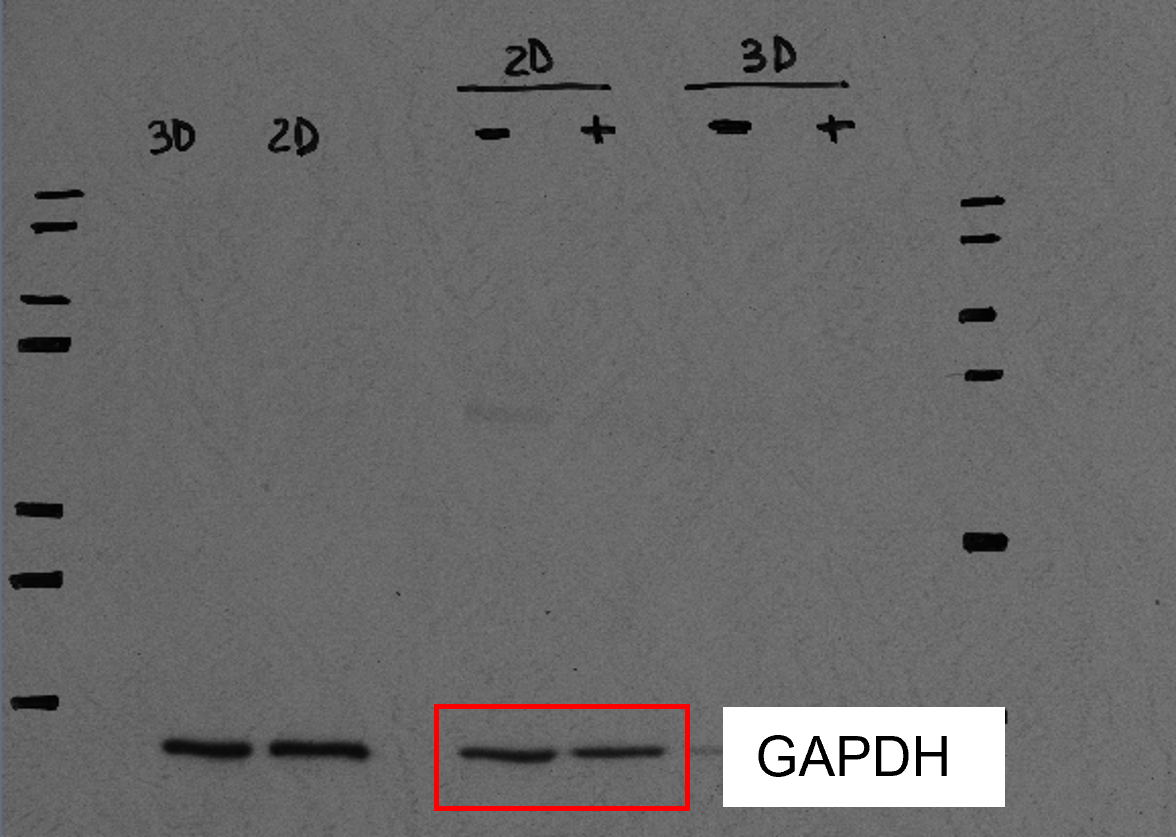

Supplement: Supplementary file 5 — Source data Fig. 5 [file 44318_2024_80_MOESM5_ESM.zip › Figure 5/5B/western_GAPDH.png]

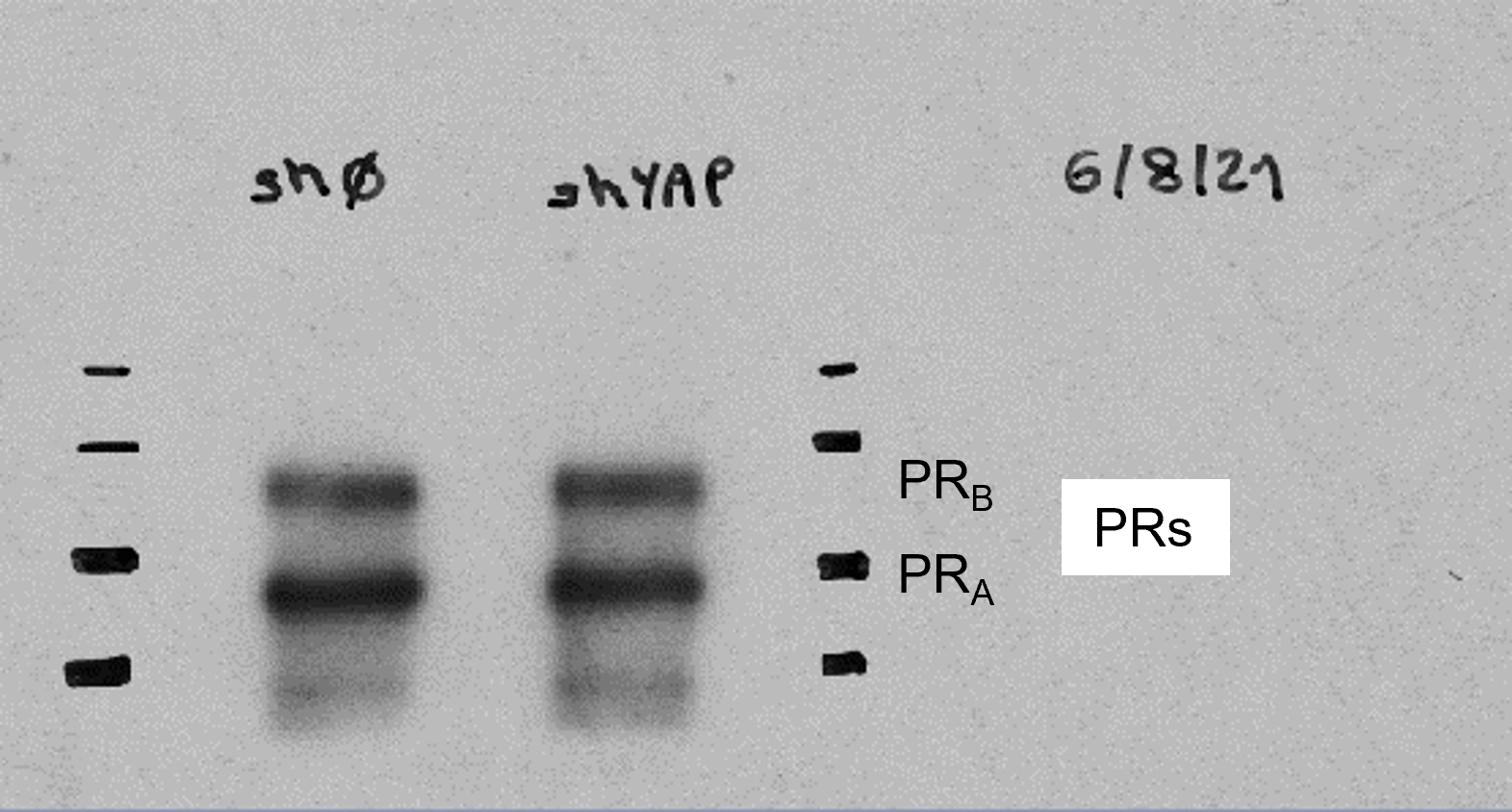

Supplement: Supplementary file 6 — Source data Fig. 6 [file 44318_2024_80_MOESM6_ESM.zip › Figure 6/6E/western_PR.png]

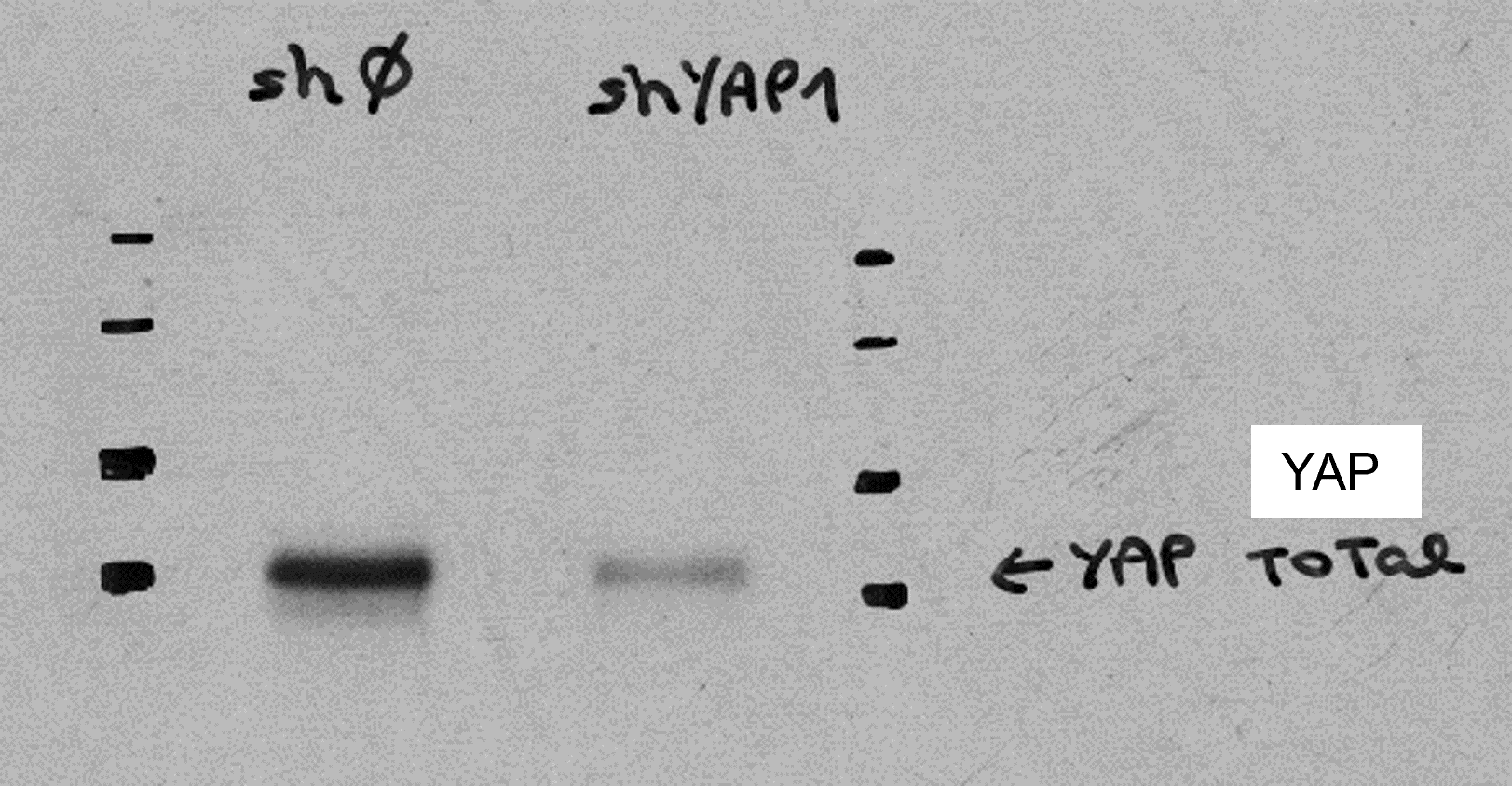

Supplement: Supplementary file 6 — Source data Fig. 6 [file 44318_2024_80_MOESM6_ESM.zip › Figure 6/6E/western_YAP.png]
