# Supplementary material for: LATS1 controls CTCF chromatin occupancy and hormonal response of 3D-grown breast cancer cells
Source: EMBO J. 2024 Apr 2;43(9):5. doi: 10.1038/s44318-024-00080-x (PMC11066098; doi:10.1038/s44318-024-00080-x)
Supplement: Supplementary file 9 — Expanded View Figures [file 44318_2024_80_MOESM9_ESM.pdf]

# Expanded View Figures

**Figure EV1. Profiles of H3K27me3, H3K9me3 and super-enhancers detected in 2D and 3D T47D breast cancer cells.**

(**A**) The H3K27me3 profiles in 3D-repressed and activated genes (blue and green lines, respectively) obtained in both conditions (first and second panels, from the left) is shown. Third and fourth panel from the left: profiles of H3K27me3 in 2D and 3D random genes. Right panel: Genome browser view of H3K27me3 ChIP-seq data in the NRCAM gene. (**B**) The H3K9me3 profiles in 3D-repressed and activated genes (blue and green lines, respectively) obtained in both conditions (first and second panels, from the left) is depicted. Third and fourth panels from the left: profiles of H3K9me3 in 2D and 3D random genes. (**C**) The enrichment of the H3K27ac signal in super-enhancers obtained from 2D and 3D cells is shown. (**D**) Venn diagram corresponding to the super-enhancers detected in T47D cells grown in 2D and 3D conditions.

 © The Author(s)

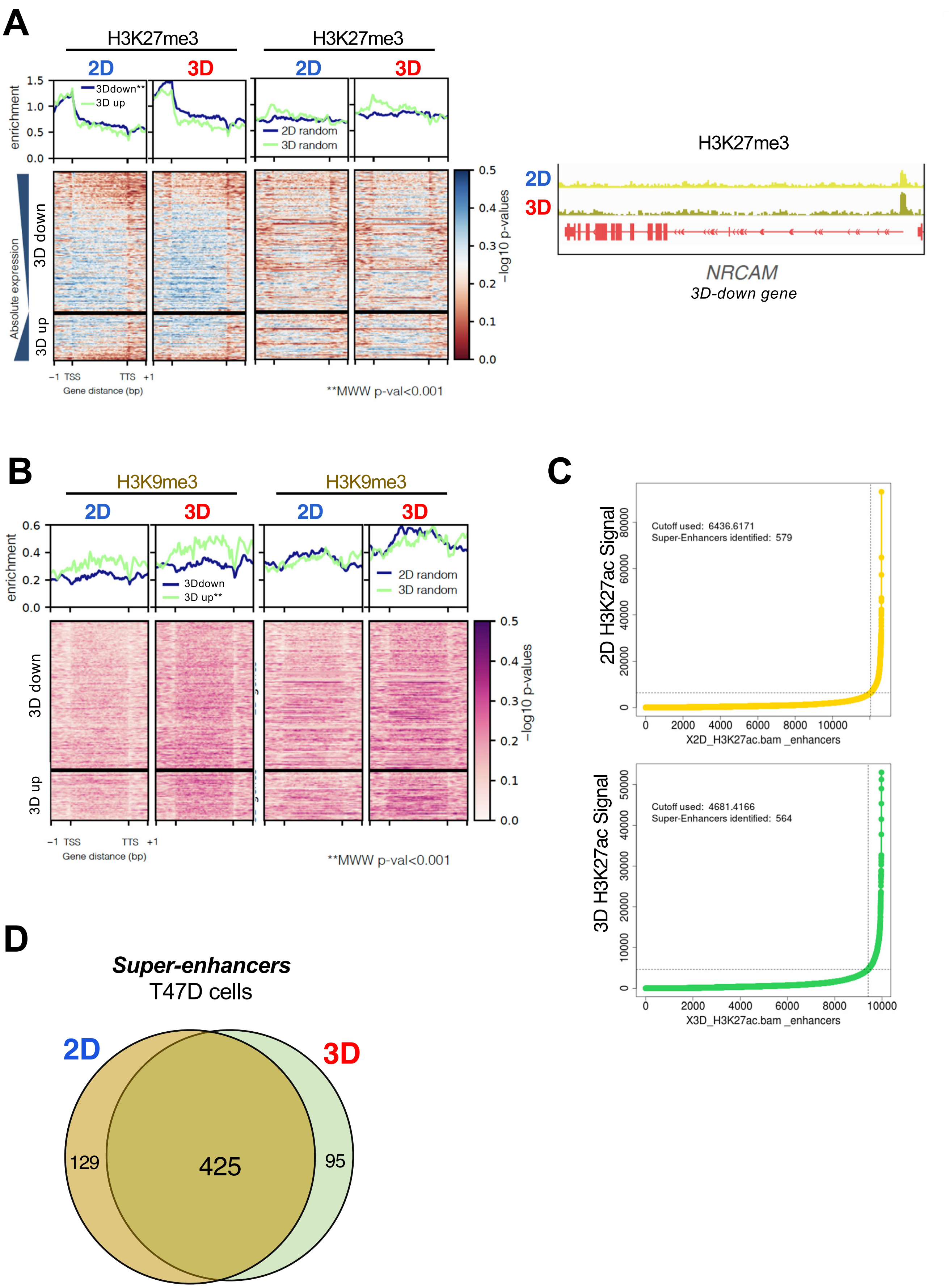
A
H3K27me3
2D
3D
H3K27me3
2D
3D
enrichment
1.5
1.0
0.5
0.0
3Ddown**
3D up
2D random
3D random
Absolute expression
3D down
3D up
−1 TSS
TTS +1
Gene distance (bp)
0.5
0.4
0.3
0.2
0.1
0.0
−log10 p-values
**MWW p-val<0.001
H3K27me3
2D
3D
NRCAM
3D-down gene
B
H3K9me3
2D
3D
H3K9me3
2D
3D
enrichment
0.6
0.4
0.2
0.0
3Ddown
3D up**
2D random
3D random
3D down
3D up
−1 TSS
TTS +1
Gene distance (bp)
0.5
0.4
0.3
0.2
0.1
0.0
−log10 p-values
**MWW p-val<0.001
C
2D H3K27ac Signal
Cutoff used: 6436.6171
Super-Enhancers identified: 579
0
20000
40000
60000
80000
0
2000
4000
6000
8000
10000
X2D_H3K27ac.bam _enhancers
3D H3K27ac Signal
Cutoff used: 4681.4166
Super-Enhancers identified: 564
0
10000
20000
30000
40000
50000
0
2000
4000
6000
8000
10000
X3D_H3K27ac.bam _enhancers
D
Super-enhancers
T47D cells
2D
3D
129
425
95

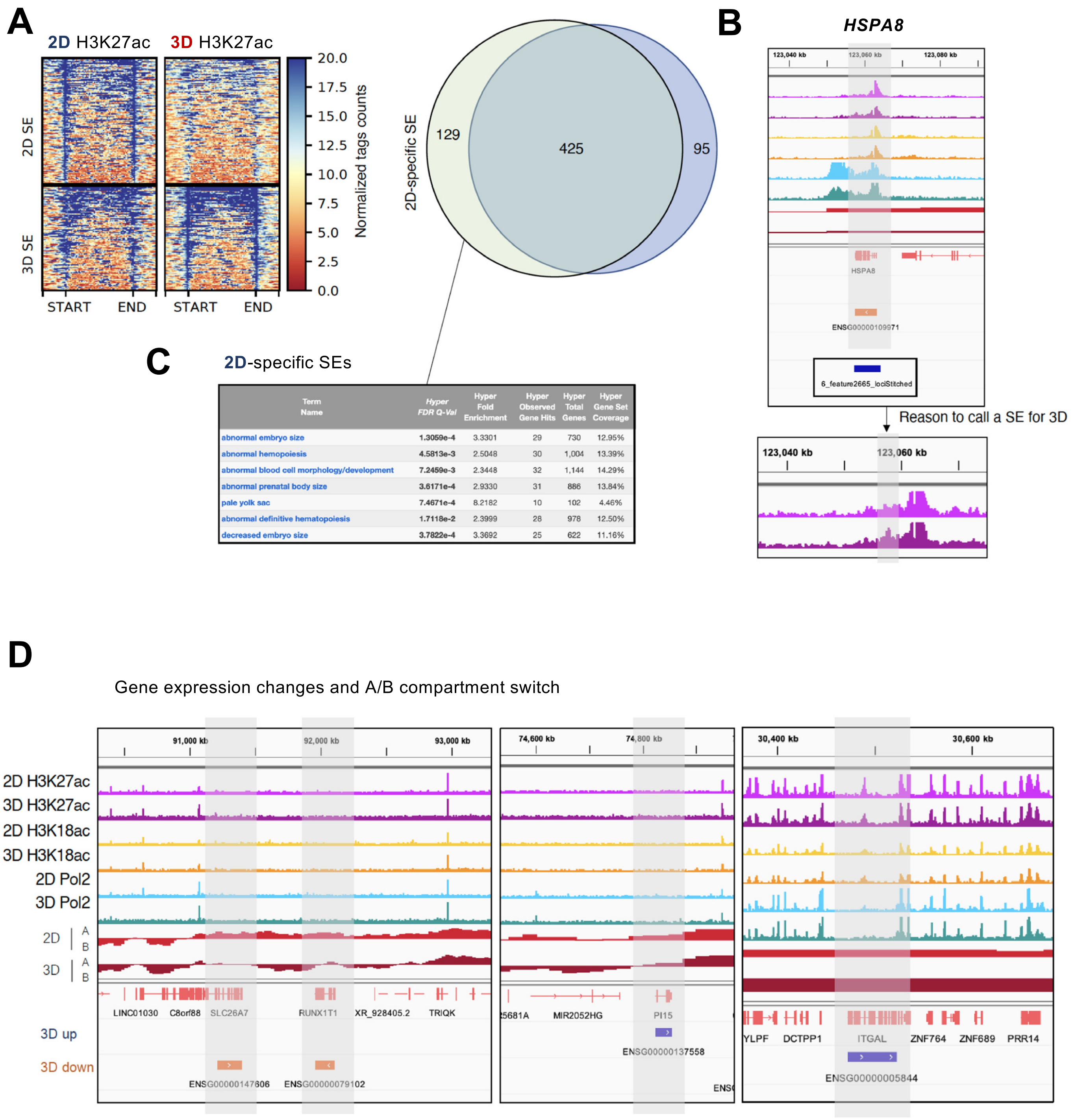


| Term Name | Hyper FDR Q-Val | Hyper Fold Enrichment | Hyper Observed Gene Hits | Hyper Total Genes | Hyper Gene Set Coverage |
|---|---|---|---|---|---|
| abnormal embryo size | 1.3059e-4 | 3.3301 | 29 | 730 | 12.95% |
| abnormal hemopoiesis | 4.5813e-3 | 2.5048 | 30 | 1,004 | 13.39% |
| abnormal blood cell morphology/development | 7.2459e-3 | 2.3448 | 32 | 1,144 | 14.29% |
| abnormal prenatal body size | 3.6171e-4 | 2.9330 | 31 | 886 | 13.84% |
| pale yolk sac | 7.4671e-4 | 8.2182 | 10 | 102 | 4.46% |
| abnormal definitive hematopoiesis | 1.7118e-2 | 2.3999 | 28 | 978 | 12.50% |
| decreased embryo size | 3.7822e-4 | 3.3692 | 25 | 622 | 11.16% |

**Figure EV2. Characterization of super-enhancers identified in 2D and 3D cells.**

(**A**) The enrichment of the H3K27ac signal in super-enhancers obtained from 2D and 3D cells is shown. Venn diagram corresponding to the super-enhancers detected in T47D cells grown in 2D and 3D conditions. By using a proximity-based script (Hnisz et al, 2013), we found 129 and 95 genes associated to 2D and 3D SEs, respectively (right panel). In the case of genes exclusively regulated in the 3D condition by SEs, many of them appear to be artifacts, as illustrated with the HSPA8 gene (**B**). (**C**) The 2D-exclusive genes are related to terms like abnormal embryo size, abnormal development, and hematopoiesis. (**D**) Snapshots from the genome browser illustrating the transitions for two 3D downregulated genes, SLC26A7 and RUNX1T1, and two 3D upregulated genes, PI15 and ITGAL are shown.

 © The Author(s)

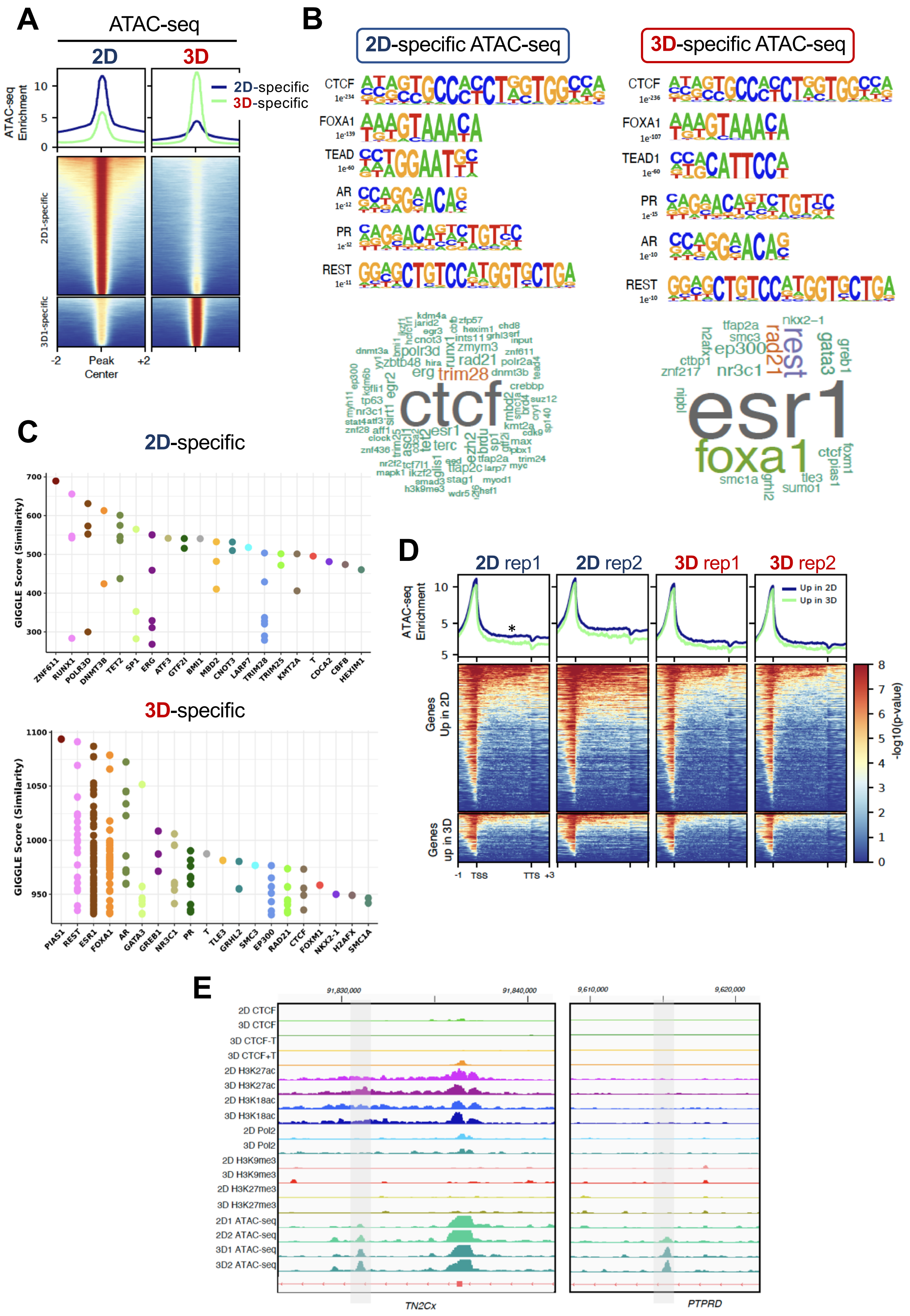
A
ATAC-seq
2D
3D
ATAC-seq Enrichment
2D-specific
3D-specific
2D1-specific
3D1-specific
-2
Peak Center
+2
B
2D-specific ATAC-seq
3D-specific ATAC-seq
CTCF
FOXA1
TEAD
TEAD1
AR
PR
REST
C
2D-specific
GIGGLE Score (Similarity)
ZNF611
RUNX1
POLR3D
DNMT3B
TET2
SP1
ERG
ATF3
GTF2I
BMI1
MBD2
CNOT3
LARP7
TRIM28
TRIM25
KMT2A
T
CDCA2
CBFB
HEXIM1
3D-specific
PIAS1
REST
ESR1
FOXA1
AR
GATA3
GREB1
NR3C1
PR
T
TLE3
GRHL2
SMC3
EP300
RAD21
CTCF
FOXM1
NKX2-1
H2AFX
SMC1A
D
2D rep1
2D rep2
3D rep1
3D rep2
ATAC-seq Enrichment
Up in 2D
Up in 3D
Genes Up in 2D
Genes up in 3D
-1
TSS
TTS
+3
-log10(p-value)
E
91,830,000
91,840,000
9,610,000
9,620,000
2D CTCF
3D CTCF
3D CTCF-T
3D CTCF+T
2D H3K27ac
3D H3K27ac
2D H3K18ac
3D H3K18ac
2D Pol2
3D Pol2
2D H3K9me3
3D H3K9me3
2D H3K27me3
3D H3K27me3
2D1 ATAC-seq
2D2 ATAC-seq
3D1 ATAC-seq
3D2 ATAC-seq
TN2Cx
PTPRD

◀ **Figure EV3. Regions more accessible in 3D are enriched in estrogenic signaling.**

(**A**) Heatmaps of ATAC-seq data performed in 2D and 3D T47D cells. The exclusive regions belonging to each condition is highlighted. (**B**) Homer motif analysis of 2D and 3D-exclusive ATAC-seq regions (upper panel). When the same regions are contrasted with available ChIP-seq data, the CTCF and ESR1 terms appear enriched (bottom panels). (**C**) Giggle score of the data presented in (**B**). (**D**) Heatmaps of the ATAC-seq signal around 2D and 3D upgenes. (**E**) Snapshot of the genome browser around TN2Cx and PTPRD genes showing the profiles of H3K27ac, RNAPol2, H3K9me3, H3K27me3 and ATAC-seq.

 © The Author(s)

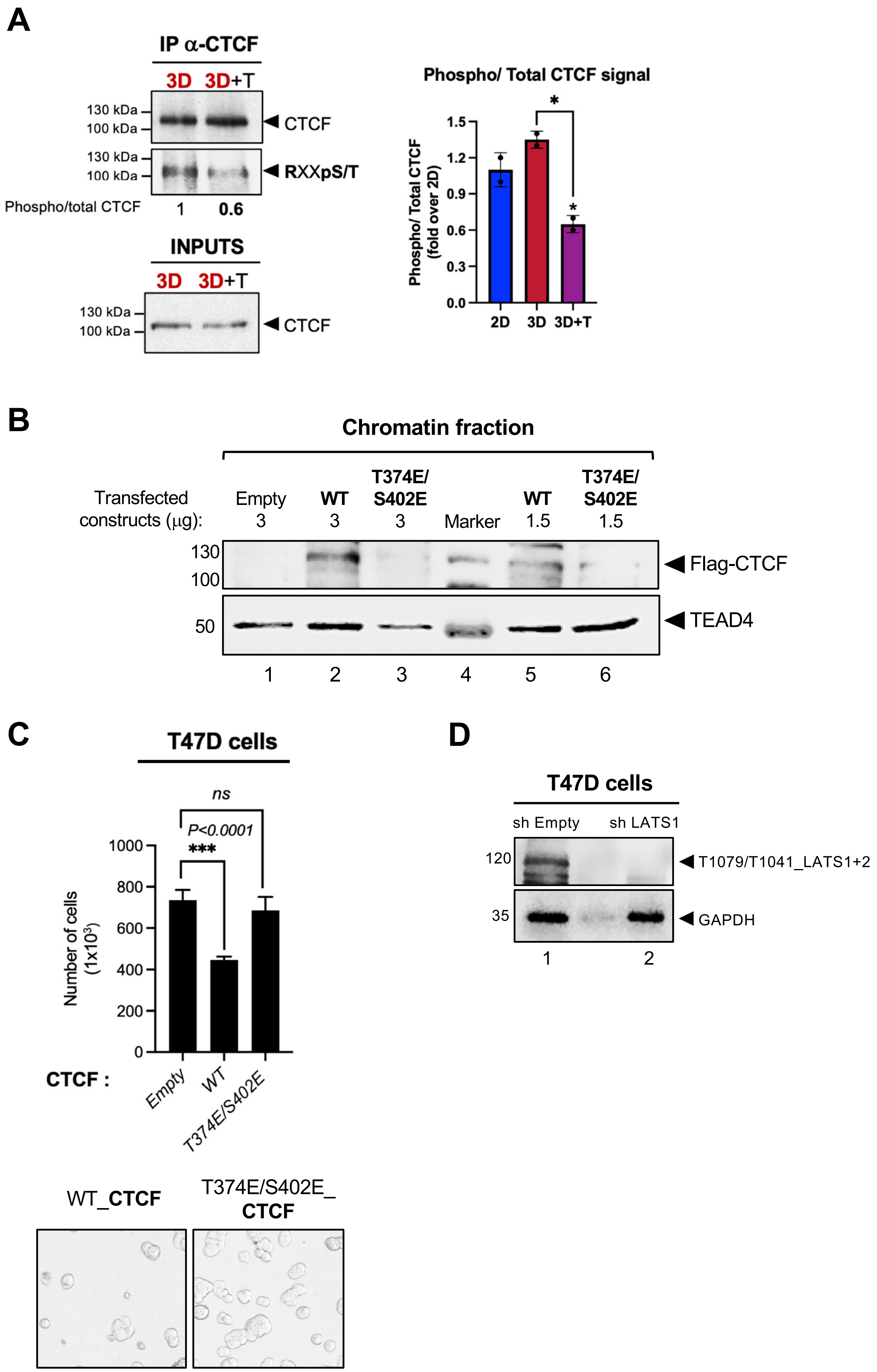
A
IP α-CTCF
3D 3D+T
130 kDa
100 kDa
CTCF
RXXpS/T
Phospho/total CTCF 1 0.6
INPUTS
3D 3D+T
CTCF
Phospho/ Total CTCF signal
Phospho/ Total CTCF (fold over 2D)
1.5
1.2
0.9
0.6
0.3
0.0
2D 3D 3D+T
B
Chromatin fraction
Transfected constructs (μg):
Empty 3
WT 3
T374E/ S402E 3
Marker
WT 1.5
T374E/ S402E 1.5
130
100
50
Flag-CTCF
TEAD4
1 2 3 4 5 6
C
T47D cells
ns
P<0.0001
***
Number of cells (1x10³)
1000
800
600
400
200
0
CTCF :
Empty
WT
T374E/S402E
WT_CTCF
T374E/S402E_ CTCF
D
T47D cells
sh Empty sh LATS1
120
35
T1079/T1041_LATS1+2
GAPDH
1 2

**Figure EV4. CTCF phosphorylation is reduced in the presence of LATS inhibitor.**

(**A**) 2D, 3D and 3D treated with TRULI T47D cells were cultured and subjected to immunoprecipitation with anti-CTCF antibodies and the immunoblotting for phospho-RxxS/T was performed. For quantification, the intensity of phospho-CTCF versus total CTCF signal in control samples is shown (right panel). (**B**) T47D cells were transiently transfected with wild-type (WT) and T374E/S402E (phospho-mimetic) CTCF flag-tagged constructs. Subsequently, cells were lysed, and the chromatin fraction was isolated to display the presence of flag-CTCF bound to the chromatin fraction. (**C**) Cell growth assays performed in T47D cells expressing both wild-type (WT) and a phospho-mimetic variant of CTCF T374E/S402E. (**D**) The levels of T1079/T1041p LATS1 + 2 signal in both shEmpty (control) and shLATS1 cells is shown. The noticeable decrease in the phospho T1079/T1041 signal and LATS1 (Fig. 5C) suggests that the predominant portion of detected p-LATS corresponds to LATS1 in the 3D model. GAPDH is used as loading control. Source data are available online for this figure.


© The Author(s)

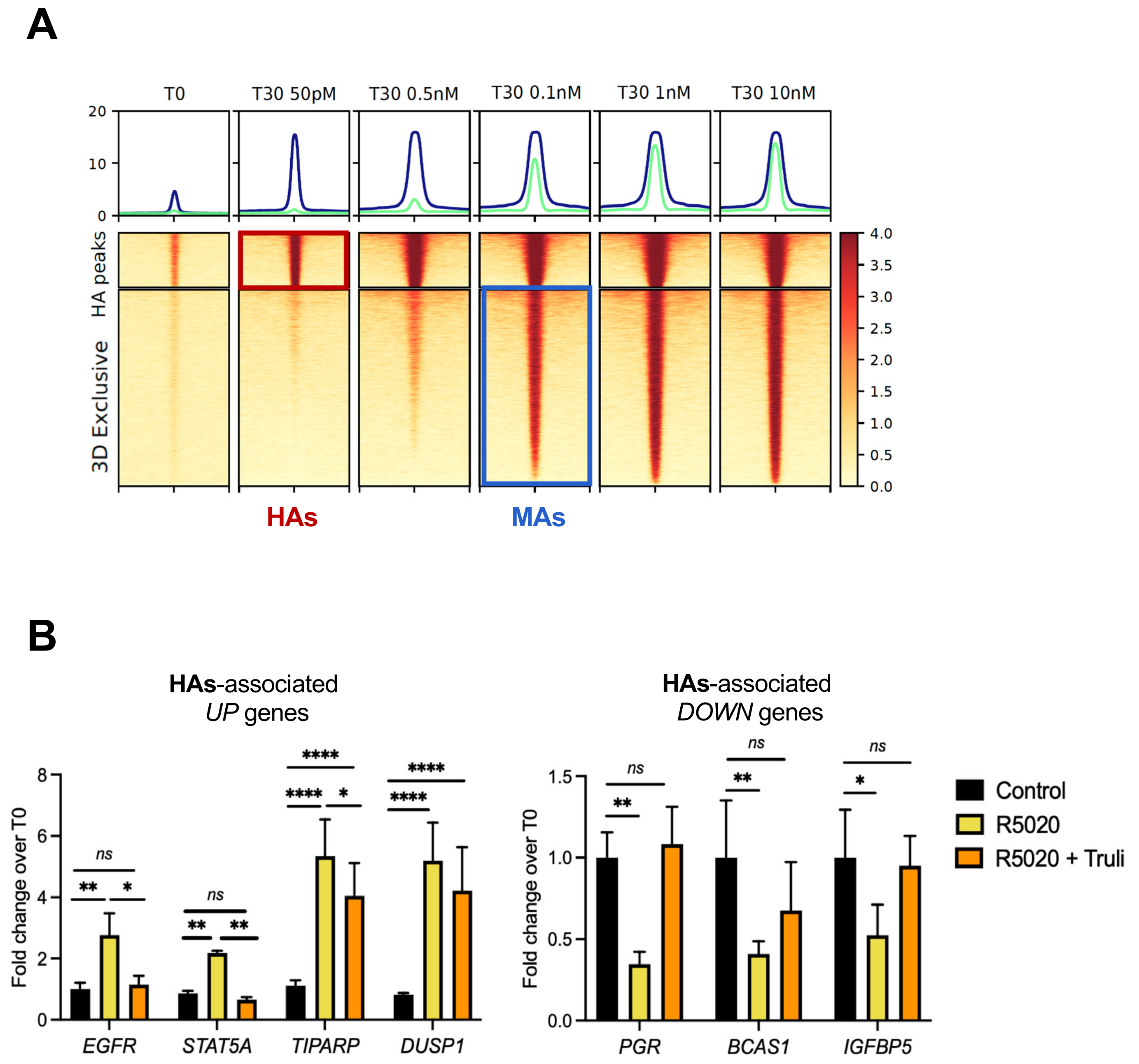


**Figure EV5. Hormone-dependent CTCF recruitment to High accessible PR binding sites (HAs) requires LATS1 activity.**

(**A**) Heatmaps of PR ChIP-seq signal obtained at different concentrations of R5020 and corresponding to HAs and 3D-exclusive PR-binding sites are shown. (**B**) Cells grown in 3D conditions and treated or not with R5020 and TRULI as indicated, were submitted to gene activity assays. Four up and three down-HAs-associated genes were tested. Results are represented as mean and SD from two experiments performed in duplicate. The *P* value was calculated using the Student's *t* test.

© The Author(s)
